# An “On–Off” AIE-Based Lock-and-Key Fluorescent Probe System for Detection of Fentanyl/Norfentanyl

**DOI:** 10.3390/molecules30091985

**Published:** 2025-04-29

**Authors:** Jing Sun, Junge Zhi, Li Zhang, Yan Qi, Jiefang Sun, Yushen Jin, Jie Yin, Kai Yao, Bing Shao

**Affiliations:** 1 National Key Laboratory of Veterinary Public Health Security, College of Veterinary Medicine, China Agricultural University, Beijing 100193, China; 2 Beijing Key Laboratory of Diagnostic and Traceability Technologies for Food Poisoning, Beijing Center for Disease Prevention and Control, Beijing 100013, China; 3 School of Chemistry and Chemical Engineering, Beijing Institute of Technology, Beijing 102488, China; 4 College of Science, China Agricultural University, Beijing 100193, China

**Keywords:** fentanyl, aggregation-induced luminescence probe, self-assembly drive, spatial specific recognition, through-space charge transfer (TSCT)

## Abstract

The misuse of fentanyl poses significant social risks, and accurately and swiftly detecting fentanyl in field settings presents a considerable challenge. Herein, we have designed and synthesized a fluorescent probe TP-CF_3_-COOH, which is composed of carboxyl- and trifluoromethyl-binding center tetraphenyl butadiene. The unique centrosymmetric configuration of the TP-CF_3_-COOH probe allows for the construction of a fluorescence “on–off” mechanism recognition platform by spatially matching fentanyl and its metabolite norfentanyl. Importantly, this study reveals that the interaction of fentanyl or norfentanyl with TP-CF_3_-COOH results in spontaneous self-assembly, generating a three-dimensional complex sphere that is smaller than the two-dimensional sheet fluorescence probe. This self-assembly process results in the quenching of fluorescence. Theoretical calculations demonstrate that this process is accompanied by intermolecular through-space charge transfer during self-assembly, leading to a blue shift in emission wavelength. As a result, the TP-CF_3_-COOH fluorescent probe enables the quantitative detection of fentanyl/norfentanyl within a range of 1 × 10^−2^–1 × 10^3^ μg/L, with limits of detection of 2 × 10^−4^ μg/L and 3 × 10^−4^ μg/L, respectively. This cost-effective, rapid, and sensitive fluorescent probe holds great potential for the onsite screening and detection of fentanyl and its analogues.

## 1. Introduction

The misuse of fentanyl and other novel psychoactive substances has emerged as a significant global issue [1,2,3]. According to the World Drug Report 2024, the opioid crisis in North America is primarily driven by fatal overdoses, with fentanyl being the leading cause of these fatalities [4,5]. About 2 mg of fentanyl can be lethal, and its rapid metabolism after ingestion produces the primary metabolite, norfentanyl [6,7,8]. Furthermore, owing to fentanyl’s highly addictive nature, its low cost, and the ease of clandestine online distribution, it has become widely abused since its introduction into the global illicit drug market [9,10]. Fentanyl, norfentanyl, and fentanyl substances have similar chemical structures, all of which are phenylpiperidine compounds mainly featuring a piperidine ring, a phenethyl group, and an amide-linked phenyl group [11]. By selecting the most representative fentanyl and its metabolite norfentanyl as analytes, the development of a rapid and accurate method for field application would undoubtedly have significant implications for exploring fluorescence-sensing methods across the entire fentanyl family.

Nowadays, methods such as chromatographic methods using various detectors and portable electrochemical sensors, surface-enhanced Raman spectroscopy, ion mobility spectrometry, immunoassay, and optical fibre have been developed for fentanyl detection [12,13,14,15,16]. At the same time, the cost of special instrumentation restricts its access to communities. Compared to these laboratory methods, the development of specific probes of fentanyl poses a significant challenge. In recent years, fluorescence detection methods have gained popularity due to their straightforward pretreatment, brief operational duration, and rapid response time [17,18,19]. Among these methods, aggregation-induced emission (AIE) luminous probes are considered particularly promising for onsite detection owing to their low cost and good sensitivity [20,21,22]. By leveraging the structural characteristics of the parent core of the AIE light source, researchers can employ molecular engineering design to recognize and analyze small molecule compounds, such as toxins, explosives, and chemical reagents [23,24,25,26].

Tetraphenylbutadiene (TPB) serves as a fundamental nuclear structure for AIE materials. Its “four-leaf spiral” facilitates the three-dimensional matching of small-molecule compounds [27,28]. The weak intermolecular interactions of the aggregates in solution are attributed to distortions in their spatial structure [29], allowing for facile self-assembly with other substances through diverse interactions, resulting in the change of fluorescence [30].

**Scheme 1:**
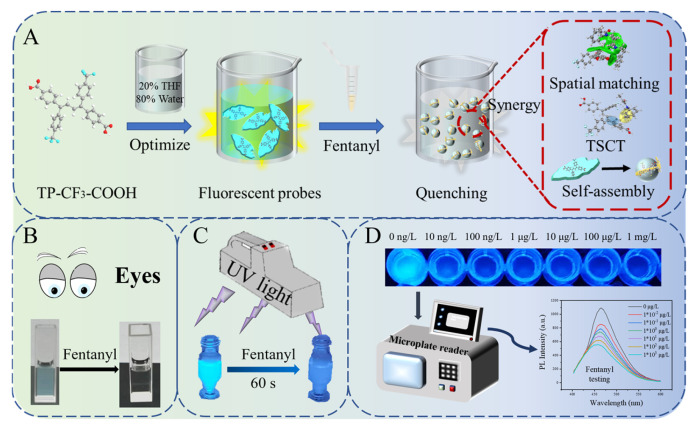
(**A**) Schematic diagram of the preparation of the TP-CF_3_-COOH probe and the application mechanism for fentanyl detection. (**B**) Visual colorimetry for qualitative detection. (**C**) UV light for qualitative detection of fentanyl. (**D**) Portable microplate reader for quantitative detection of fentanyl.

In this study, we synthesized four types of three-dimensional symmetric AIE fluorescent probes based on the core structure of 1,1,4,4-tetraphenylbutadiene. We identified dimethyl 4,4′-((1Z,3Z)-1,4-bis(4-(trifluoromethyl)phenyl)butane-1,3-diene-1,4-diyl)dibenzoic acid (TP-CF_3_-COOH) as the probe for the detection of fentanyl. Incorporating trifluoromethyl and carboxyl ligands confers favourable biocompatibility, cost-effectiveness, ease of storage, and direct detection to the fluorescence probe. Initially, the TP-CF_3_-COOH solution probe demonstrated pronounced aggregation-induced luminescence; however, it transitioned to an ‘on–off’ mode following the introduction of fentanyl/norfentanyl. The fluorescent probe can selectively recognize fentanyl/norfentanyl through weak electrostatic interactions, hydrogen bonding, and dispersion forces to form a unique three-dimensional spatial assembly. Employing visualization techniques, thermodynamic monitoring, and theoretical calculations, we observed a synergistic coupling between self-assembly-driven conformational changes and intermolecular through-space charge transfer (TSCT) during the recognition process with fentanyl/norfentanyl. This coupling leads to a significant attenuation in fluorescence intensity and a blue shift (Scheme 1A). Moreover, TP-CF_3_-COOH solution fluorescent probes can facilitate the direct qualitative detection of trace amounts of fentanyl with the naked eye (Scheme 1B) or ultraviolet (UV) light (Scheme 1C). The portable microplate reader also enables the quantitative analysis of fentanyl in aqueous solutions (Scheme 1D).

## 2. Results

### 2.1. Synthesis and Characterization

**Figure 1:**
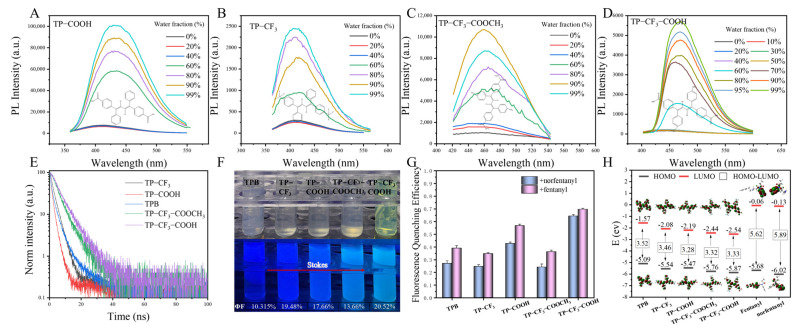
PL intensity of (**A**) TP-COOH, (**B**) TP-CF_3_, (**C**) TP-CF_3_-COOCH_3_, and (**D**) TP-CF_3_-COOH at different water contents. (**E**) Fluorescence lifetime of 5 solid-state AIE materials. (**F**) Digital images and fluorescence quantum yields of TPB, TP-COOH, TP-CF_3_, TP-CF_3_-COOCH_3_, and TP-CF_3_-COOH. (**G**) Fluorescence quenching efficiency of 5 AIE materials (100 μg/L) in a mixture of THF/water (1:9, *v*/*v*) and separately added fentanyl/norfentanyl (10 μg/L). (**H**) Energy levels of different substances calculated by B3LYP/6-31G (d, p).

We synthesized four TPB compounds with different groups. TPB serves as the core structure for coupling the bridge and fluorophore in these compounds, while the -CF_3_, -COOH, and -COOCH_3_ groups impart hydrophobic and hydrophilic properties, respectively. These substituents collectively enhance the recognition capability of fentanyl/norfentanyl-binding carriers [31,32]. To analyze the AIE properties of TPB and its four derivatives, the fluorescence (FL) emission spectra of these substances in THF/water mixtures with different water fractions (fw) at optimum excitation wavelengths are presented in Figure S5 and Figure 1A–D. Notably, when fw exceeded 60%, the FL intensities exhibited a significant enhancement, whereas the FL intensities increased only slightly when fw was below 60%.

Additionally, as depicted in Figure 1E, multi-colour luminescence ranging from blue to green indicates that different substituents are crucial in generating long-wavelength emissions. This observation is consistent with Table S1, which shows that TP-CF_3_-COOH exhibits a superior emission shift by approximately 470 nm. In applying fluorescent probes, a more significant Stokes shift avoids emission wavelength interference and a poor signal-to-noise ratio [33]. The longer lifetime (Figure 1E) and higher absolute quantum yield (Figure 1F) of TP-CF_3_-COOH can be attributed to the restriction of the rotation of the TPB parent nucleus by the -CF_3_ and -COOH groups. To evaluate the interaction capabilities of the five substances with fentanyl/norfentanyl, binding experiments were performed by adding fentanyl/norfentanyl to the identical concentrations of the five substances. The PL spectra (Figure 1G and Figure S6) show that all substances exhibited distinct fluorescence quenching effects, especially TP-CF_3_-COOH, demonstrating the highest quenching efficiency of 80%. We postulate that the combined presence of the -CF_3_ and -COOH moieties synergistically enhances the binding affinity towards fentanyl/norfentanyl, thereby intensifying fluorescence quenching. This observation aligns well with our theoretical calculations presented in Table S2, where TP-CF_3_-COOH + fentanyl/norfentanyl exhibits the most favourable binding free energy of −15.41 kJ/mol and −15.08 kJ/mol, respectively.

Furthermore, the electronic structures of the five substances were compared with those of fentanyl and norfentanyl using density functional theory (DFT) calculations performed by the B3LYP/6-31G (d,p) method. As demonstrated in Figure 1H, compared with fentanyl/norfentanyl, the AIE material exhibits a similar HOMO energy level but a lower LUMO energy level. This suggests that it may act as a receptor in the reaction process [34,35]. The molecular structures of fentanyl and norfentanyl are illustrated in Scheme S2. Within these compounds, the amide oxygen atom, amide nitrogen atom, and piperidine nitrogen atom serve as the principal interaction sites for fentanyl analogues in biological systems. Recent studies have indicated that fentanyl potentially leads to charge transfer as a donor in biological systems [36,37]. Consequently, it can be inferred that fentanyl/norfentanyl may act as the donor while the five substances serve as acceptors, resulting in charge transfer upon their combination.

Based on the results of the aforementioned experiments, TP-CF_3_-COOH, exhibiting superior optical properties and remarkable FL quenching efficiency, was selected for further research on constructing a fluorescent turn-off sensor [38]. As illustrated in Figure S7A, the FL quenching efficiency is the highest when the fw in the THF/water mixtures reaches 80%. The PL intensity of the TP-CF_3_-COOH fluorescence probe remains stable after ultrasonication (Figure S7B). Furthermore, other experimental (Figure S7C–F) findings demonstrated the TP-CF_3_-COOH probe solution’s excellent resting stability, temperature stability, serum stability, and wide pH tolerance range. Consequently, the optimized probe was chosen for subsequent experiments. An X-ray diffraction (XRD) analysis (Figure S8) revealed that the TP-CF_3_-COOH belonged to the P21/c (14) spatial group in a monoclinic crystal system. A thermogravimetric analysis (TGA) of TP-CF_3_-COOH was carried out by TGA testing, and the result obtained (Figure S9) indicates its remarkable thermal stability with no appreciable weight loss until 150 °C.

### 2.2. Probe Recognition Mechanism

A detection platform based on molecular recognition and matching lies in the spontaneous recognition process supported by thermodynamics, the identification of specific binding sites between target molecules and test molecules, and the occurrence of stable intermolecular interactions accompanied by signal changes driven by energy alterations. Therefore, a more comprehensive experimental exploration is required in order to gain a clearer understanding of the molecular recognition and matching process between TP-CF_3_-COOH and fentanyl/norfentanyl [39].

**Figure 2:**
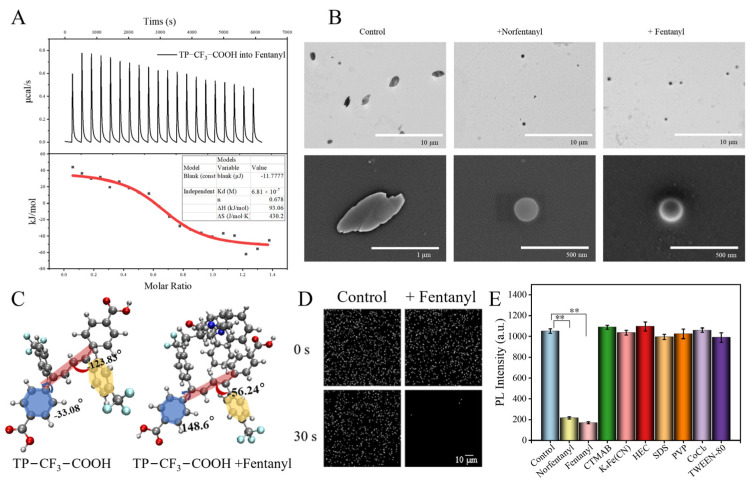
ITC of (**A**) TP-CF_3_-COOH into fentanyl. (**B**) TEM and SEM of TP-CF_3_-COOH probe, TP-CF_3_-COOH + norfentanyl, and TP-CF_3_-COOH + fentanyl. (**C**) The conformation of the ground state of TP-CF_3_-COOH and TP-CF_3_-COOH + fentanyl. (**D**) HCI of fluorescence quantum points within 30 s. Conformation of TP-CF_3_-COOH after binding fentanyl. (**E**) Fluorescence intensity of TP-CF_3_-COOH probe in different surfactants and quenchers (Control, Norfentanyl, Fentanyl, Cetyltrimethylammonium bromide (CTMAB), K_4_Fe (CN), Hydroxyethyl Cellulose (HEC), Sodium Dodecyl Sulfate (SDS), Polyvinyl Pyrrolidone (PVP), CoCl_2_, and TWEEN-80). ** indicates *p* < 0.01.

Firstly, the thermodynamic parameters such as binding affinity, enthalpy change, and entropy change were determined through the monitoring of the dynamic heat exchange process during the identification process (Figure 2A, Figures S10 and S11). After subtracting the background heat of titration, the experimental results for TP-CF_3_-COOH + fentanyl/norfentanyl showed ΔH > 0 and ΔS > 0, indicating that this is an endothermic process accompanied by a significant increase in entropy. This may be attributed to changes in the molecular conformation of TP-CF_3_-COOH upon binding fentanyl/norfentanyl, along with an entropy-driven process dominated by hydrophobic interactions. According to the formula ΔG = ΔH-TΔS, negative ΔG values (−35.14 kJ/mol for TP-CF_3_-COOH + fentanyl and −35.46 kJ/mol for TP-CF_3_-COOH + norfentanyl) suggest that both binding processes are spontaneous and form a relatively stable matching structure. However, some discrepancies exist between the experimental results obtained through isothermal titration calorimetry (ITC) and the Gibbs free energy calculated using DFT (Table S2). The possible reason is that computational chemistry only considers a single-solution background. In contrast, the ITC experiment involves a mixed solution, leading to a larger absolute value of ΔG, indicating a closer interaction between the two systems. Additionally, a molecular structure analysis was performed on TP-CF_3_-COOH before and after binding, revealing significant changes in the dihedral angles (Figure 2C and Figure S12). These findings suggest that the addition of fentanyl/norfentanyl alters the molecular conformation of TP-CF_3_-COOH, resulting in increased intramolecular rotation and a hindered luminescence performance of AIE. These observations are consistent with the ITC results.

Subsequently, to visually analyze the binding process of the self-assembly, various experiments, including transmission electron microscope (TEM), scanning electron microscope (SEM), and high-content imaging (HCI), were employed [40]. The TEM and SEM images presented in Figure 2B demonstrate that the TP-CF_3_-COOH probe monomer exists as two-dimensional nanosheets with a size of 0.87 μm in the mixed solution. After the addition of fentanyl or norfentanyl, a small three-dimensional spherical structure is formed. Intriguingly, subsequent HCI experiments depicted in Figure 2D, Figure S13, and Video S1 (control group) and Video S2 (+fentanyl group), reveal a stepwise quenching of fluorescence exhibited by the fluorescence probe during the 30 s contact process between TP-CF_3_-COOH and fentanyl/norfentanyl. Based on the experiments mentioned above, it can be deduced that the robust binding affinity between TP-CF_3_-COOH and fentanyl/norfentanyl has the capability to disrupt the aggregate structure of the aggregation-induced probe itself, leading to the spontaneous formation of spherical self-assembly with a more intricate three-dimensional architecture. To elucidate the specific binding process, it is imperative that we introduce additional substances capable of decomposing the polymer into the fluorescence probe and to conduct further analysis of the changes in the fluorescence signal.

We examined several surfactants and quenchers as comparative substances by adding them to the TP-CF_3_-COOH probe needle solution (Figure 2E). The results indicate that, unlike the fentanyl/norfentanyl-induced fluorescence quenching intensity observed earlier, other substances potentially affecting the AIE material aggregation state do not exhibit such a phenomenon. This observation highlights the unique affinity between TP-CF_3_-COOH and fentanyl/norfentanyl self-assembly.

### 2.3. Multiple Specific Interactions Between Probes and Fentanyl/Norfentanyl

To verify the specific recognition of TP-CF_3_-COOH towards fentanyl/norfentanyl, a fluorescence quenching selectivity experiment was conducted. Fentanyl and norfentanyl were used as target analytes, while two structurally similar compounds (compound **1**, *N*-phenylpropionamide; and compound **2**, *N*-cyclohexyl-*N*-phenylpropionamide) and multiple drugs (heroin, cocaine, ketamine, cathinone, and amphetamine) served as control substances. As shown in Figure S14, TP-CF_3_-COOH demonstrated significant selectivity towards fentanyl/norfentanyl, evidenced by the notable fluorescence quenching, whereas negligible quenching occurred with the control substances. This specific fluorescence quenching can be attributed to the formation of a tightly bound self-assembly between TP-CF_3_-COOH and fentanyl/norfentanyl. To elucidate the specific recognition mechanism of TP-CF_3_-COOH towards fentanyl/norfentanyl, an in-depth analysis of the strong interaction within the self-assembly is necessary.

**Figure 3:**
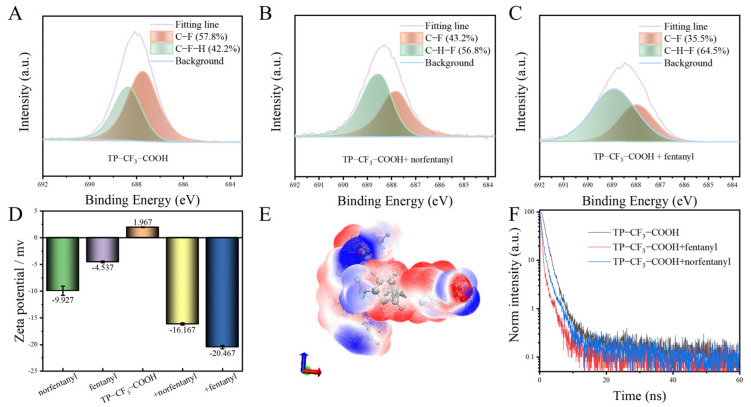
Fluorescence changes after adding (**A**) XPS high-resolution F1s spectra of TP-CF_3_-COOH probe, (**B**) TP-CF_3_-COOH + fentanyl, and (**C**) TP-CF_3_-COOH + norfentanyl. (**D**) Zeta potential of norfentanyl, fentanyl, TP-CF_3_-COOH probe, TP-CF_3_-COOH + fentanyl, and TP-CF_3_-COOH + norfentanyl. (**E**) The surface penetration of the electrostatic potential of TP-CF_3_-COOH + fentanyl. (**F**) The fluorescence lifetime of TP-CF_3_-COOH probe, TP-CF_3_-COOH + fentanyl, and TP-CF_3_-COOH + norfentanyl.

A ^1^H NMR (Figure S15) analysis indicated no formation of new species in the TP-CF_3_-COOH + fentanyl/norfentanyl complex, but the disappearance of active hydrogen at 11 ppm suggested potential intermolecular hydrogen bonding interactions. X-ray photoelectron spectroscopy (XPS) experiments (Figure S16 and Figure 3A–C) demonstrated a significant increase in C-H-F bond content after adding fentanyl/norfentanyl, indicating enhanced intermolecular H-F bonding [41,42]. Conversely, a decrease in C-F bond content suggested a disruption of the intramolecular hydrogen bonding structure within the TP-CF_3_-COOH monomer, leading to the accelerated rotation of its parent nucleus and subsequent fluorescence intensity reduction.

The zeta potentials of norfentanyl, fentanyl, TP-CF_3_-COOH probe, TP-CF_3_-COOH + fentanyl, and TP-CF_3_-COOH + norfentanyl were measured for an analysis of their close binding effects (Figure 3D). These results indicate that the combination of negatively charged fentanyl/norfentanyl with positively charged TP-CF_3_-COOH leads to self-assembly with a higher absolute zeta potential, indicating better dispersibility and stability. Based on these findings, intermolecular van der Waals surface penetration maps were generated to visualize the electrostatic potential coloring of the self-assembly TP-CF_3_-COOH + fentanyl (Figure 3E and Figure S17) and TP-CF_3_-COOH + norfentanyl self-assemblies (Figure S18). These maps demonstrate significant penetration between fentanyl/norfentanyl and TP-CF_3_-COOH, with the red regions (positive electrostatic potential) penetrating blue regions (negative electrostatic potential). The mutual penetration distances between fentanyl/norfentanyl and TP-CF_3_-COOH are calculated as 2 × 0.31–2 × 0.9 Å and 2 × 0.4–2 × 0.95 Å, respectively. Multiple sites’ overlapping red and blue regions demonstrate mutually complementary electrostatic potential and attraction interactions.

The possible fluorescence quenching mechanism during the self-assembly formation was further discussed. Based on the PL lifetimes (τ) of the TP-CF_3_-COOH probe, TP-CF_3_-COOH + fentanyl, and TP-CF_3_-COOH + norfentanyl, which are approximately 1.19 ns, 0.25 ns, and 0.53 ns, respectively (Figure 3F), it was observed that these values for self-assembly are significantly shorter than those of the monomeric TP-CF_3_-COOH. This observation aligns well with the through-space charge transfer (TSCT) emission properties. Moreover, the reduction in fluorescence lifetime within the self-assembly suggests that the internal filtering effect is not responsible for fluorescence quenching [43]. Following the interaction of the probe with fentanyl and norfentanyl, the UV spectra (Figure S19) demonstrated the occurrence of direct binding [44].

### 2.4. Theoretical Calculation

**Figure 4:**
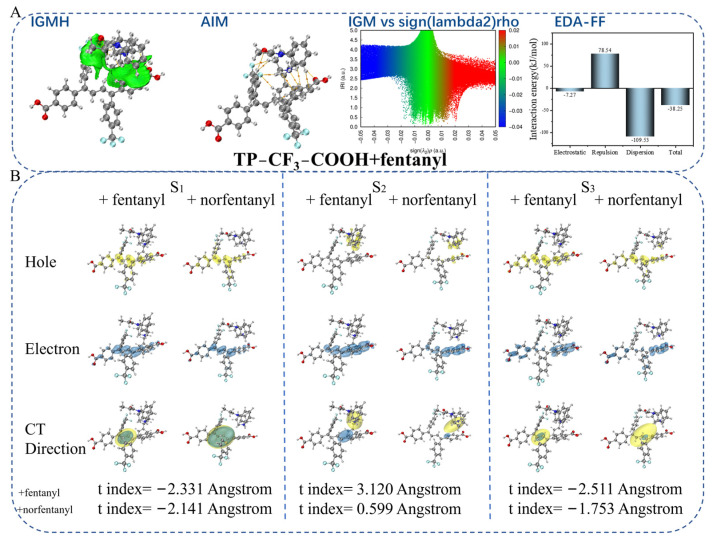
Theoretical and computational chemistry. (**A**) Interaction force analysis of TP-CF_3_-COOH + fentanyl, including IGMH, AIM, sign (lambda2) rho, and EDA-FF. (**B**) Hole–electron transfer diagram of TP-CF_3_-COOH + fentanyl and TP-CF_3_-COOH + norfentanyl.

CAM was employed to optimize the self-assembly conformation, followed by a time-dependent (TD) DFT calculation to understand the self-assembly formation mechanism fully. An independent gradient model based on Hirshfeld partition (IGMH) visual analysis (Figure 4A-IGMH and Figure S20-IGMH) displayed a continuous green isoplane between the TP-CF_3_-COOH probe and fentanyl/norfentanyl, indicating extensive and robust intermolecular interactions. An atom–molecular topological analysis quantitatively characterized these interactions, marking attractive pairs of atoms with orange spheres denoting intermolecular gravitation and lines representing bond paths at bond critical points (BCP) (Figure 4A-BCP and Figure S20-BCP). Eleven independent BCP were identified between TP-CF_3_-COOH + fentanyl molecules, while eight BCP were found between TP-CF_3_-COOH + norfentanyl molecules, signifying multiple medium-strength hydrogen bonds within the self-assembly. A strong face-to-face aromatic π–π stacking interaction and moderate van der Waals interaction existed between both molecules. Enhanced non-covalent van der Waals interactions were evident in the independent gradient model (IGM) visualization (Figure 4A-IGM and Figure S20-IGM). The energy decomposition analysis based on a forcefield (Figure 4A-EDA-FF and Figure S20-EDA-FF) method calculated an interaction energy of −38.25 kJ/mol between fentanyl and TP-CF_3_-COOH, with dispersion playing a decisive role in self-assembly binding, contributing as much as −109.53 kJ/mol, while electrostatic effects contributed a small amount (−7.27 kJ/mol). The exchange repulsion partially counteracts the electrostatic and dispersive attractions, primarily driven by significant π–π packing and molecular deformation between both molecules. Thus, dispersion forces are believed to play a fundamental role in this process [45]. Simultaneously, the dispersion force correlates with the molecular deformability, meaning a higher degree of deformability results in a stronger dispersion force. Overall, the interaction between these molecules is predominantly governed by the dispersion force, complemented by the electrostatic force, collectively driving the formation of self-assembly.

Subsequently, the electron–hole distribution in the excited state of the self-assembly and its corresponding separation coefficient (t index), representing the degree of hole–electron separation, were computed (Figure 4B). Taking the self-assembly of TP-CF_3_-COOH + fentanyl as an example, it was observed that both holes (blue region) and electrons (green area) were located on the conjugated surface of the self-assembly, with a t index value of −2.331 Å. This suggests that the S_1_ state in the dimer represents a typical location-excited (LE) state dominated by the self-assembly. Furthermore, in the S_2_ state, electrons and holes are distributed parallel to the respective conjugate surfaces of self-assembly, leading to a charge transfer from fentanyl to TP-CF_3_-COOH. The calculated t-index value is 3.120 Å, indicating an electron transfer amount of 0.98 (Table S3) and revealing the intermolecular TSCT property associated with the S_2_ state. Although some hole distribution occurs near the structure of fentanyl in the S_3_ state, the overlapping distribution between the electron holes results in a negative t-index value (−2.511 Å), signifying another LE state for S_3_.

Specifically, the atomic (Figures S21 and S22) contribution value is visualized in a heat map and complemented with Tables S3–S6. The voids observed in the heat map suggest that the primary influence on the hole in the S_2_ state (red matrix element) is predominantly attributed to the nitrogen atom of the piperidine ring in fentanyl, contributing 63.1% of the total effect. The remaining fractions are primarily attributed to the diene group in TP-CF3-COOH. However, other regions also make non-negligible contributions, resulting in a blue colouration within certain areas beyond the 25–35 range on the heat map. The overlap between heat maps suggests a minimal spatial coincidence between electrons and holes within the system region. Conversely, a significant overlap occurs between electrons and holes in both S_1_ and S_3_ states, as depicted by their respective heat maps.

Furthermore, similar computational studies were conducted on two fentanyl analogues (3-methylfentanyl and ocfentanyl) using TP-CF_3_-COOH as the binding molecular. The results (Figures S23 and S24, Tables S7 and S8) demonstrate that the two analogues generally exhibit intermolecular TSCT phenomena at their S_2_ state with a high spatial congruence, leading to fluorescence quenching and emission wavelength blue shift effects. Therefore, it has been theoretically substantiated that the strong interaction of fentanyl with TP-CF_3_-COOH involves hydrogen bonding, electrostatic forces, and dispersion forces while facilitating intermolecular TSCT, which is the fundamental mechanism underlying the specific recognition between the fentanyl and TP-CF_3_-COOH.

The fluorescence blue shift mechanism, resulting from the quenching of self-assembly and space charge transfer, was further explored (Figure S25). In the THF/H_2_O solvent, a stable self-assembly can form between TP-CF_3_-COOH and fentanyl due to a significantly stronger self-assembly driving force compared to the aggregation force provided by the low-solubility solvent, resulting in fluorescence quenching. However, despite the environmental resistance surpassing the self-assembly driving force in the mixed solution of silicone oil and water, intermolecular charge transfer can occur between them, leading only to a fluorescence blue shift without a significant quenching phenomenon.

**Figure 5:**
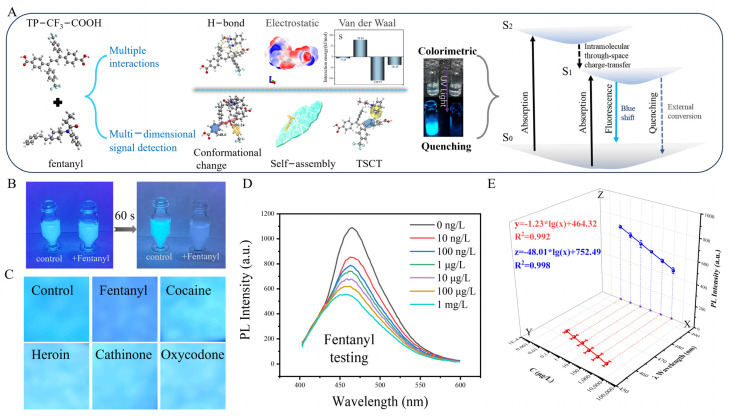
(**A**) Mechanism of specific recognition of TP-CF_3_-COOH towards fentanyl. (**B**) Digital image depicting the alteration in the solution vial subsequent to the addition of fentanyl. (**C**) Detection of 5 different kinds of drugs by TP-CF_3_-COOH-based test paper. (**D**) Quantification of fentanyl in aqueous solution. (**E**) Calibration curve for fentanyl detection.

Based on the multidimensional experiments and computational chemistry methods described above, we have determined that fentanyl/norfentanyl and TP-CF_3_-COOH are intricately linked through various forces, such as hydrogen bonding, electrostatic interaction, and dispersion action. This connection forms a specific detection mechanism involving spatial key recognition. The phenomena of fluorescence quenching and emission wavelength displacement can be understood through several mechanisms: changes in molecular conformation within the AIE fluorescence group, changes in the formation of three-dimensional self-assemblies, and the space charge transfer in the S_2_ state. These mechanisms cause colorimetric properties and fluorescence quenching (Figure 5A).

### 2.5. Analysis of Samples

The impressive performance of TP-CF_3_-COOH prompted us to explore its potential in fentanyl/norfentanyl detection involved in drug-related crimes. We initially conducted a visual qualitative detection of fentanyl in solution. As depicted in Figure 5B, 50 μg/L of fentanyl was added to the TP-CF_3_-COOH solution reaction for 60 s, and the quenched fluorescence was observable to the naked eye. Furthermore, we immersed a piece of filter paper (1 cm × 1 cm) into 1 × 10^4^ μg/L TP-CF_3_-COOH solution for 30 s. After vacuum drying, we obtained AIE fluorescence test paper. We then applied 10 μL aqueous solution (1000 μg/L) of fentanyl/cocaine/heroin/cathinone/oxycodone individually onto the test paper. Under the irradiation of a 365 nm UV lamp, the fluorescence change was captured using a mobile phone camera. Only the paper treated with fentanyl exhibited a distinct blue fluorescence, while the other drugs showed a negligible fluorescence change (Figure 5C). The phenomenon may be attributed to the occurrence of TSCT between TP-CF_3_-COOH and fentanyl during the binding interaction. However, the solid paper substrate may hinder the self-assembly of the probe and molecular conformational change, negatively impacting the quenching results and resulting solely in an emission wavelength blue shift. This conclusion is consistent with the experimental results presented in Figure S25.

Subsequently, leveraging the specific binding affinity between TP-CF_3_-COOH and fentanyl/norfentanyl, we developed a selective fluorescence detection method for fentanyl in water and norfentanyl in serum, respectively. As illustrated in Figure 5D,E and Figure S26, the fluorescence intensity exhibited a negative correlation with the concentration of the analyte, while the degree of the emission wavelength blue shift showed a positive correlation with the analyte concentration. The linear relationship between the concentration and fluorescence intensity was established as the quantitative equation (fentanyl: z = −48.01(±1.02) × lg(x) + 752.49(±3.83), norfentanyl: z = −103.05(±3.21) × lg(x) + 1240.20(±11.43)), whereas the relationship between the emission wavelength shift and concentration served as an auxiliary qualitative equation (fentanyl: y = −1.23(±0.06) × lg(x) + 464.32(0.11), norfentanyl: y = −2.01(±0.17) × lg(x) + 467.07(±0.08)). All the equations showed a good linearity with linear regression coefficients (R^2^) above 0.99. The detection limits were determined to be 2 × 10^−4^ μg/L for fentanyl in water and 3 × 10^−4^ μg/L for norfentanyl in serum using the 3σ/S formula [46]. Furthermore, the results obtained using the TP-CF_3_-COOH probe method showed excellent agreement with those from the HPLC-MS/MS method, as detailed in Tables S9 and S10. As shown in Table S11, compared with previously reported methods [47,48,49,50,51], the novel method displayed a high sensitivity, as well as a comparatively broad linear range.

The diversity of fentanyl compounds poses challenges for the rapid detection of the entire class of substances. Fortunately, the fluorescence probe has demonstrated the capacity to detect other fentanyl analogues. As illustrated in Figure S27, the addition of solutions of fentanyl analogues (1 μg/L) in the TP-CF_3_-COOH fluorescent probe resulted in fluorescence shifts of varying degrees, corresponding to fluorescence quenching of varying intensities. Different fentanyl analogues possess distinct functional groups, leading to varying degrees of TSCT and, consequently, different emission wavelength shifts. This characteristic offers excellent potential for the accurate qualitative and quantitative detection of various fentanyl analogues.

## 3. Materials and Methods

### 3.1. Materials

Detailed information regarding the reagents, instruments, and equipment are provided in Supplementary Materials.

### 3.2. Synthesis of Probes

Four TPB derivatives with different functional groups, namely, (1E,3E)-1,4-diphenyl-1,4-bis(4-(trifluoromethyl)phenyl) buta-1,3-diene (TP-CF_3_), 4,4′-((1E,3E)-1,4-diphenylbuta-1,3-diene-1,4-diyl)dibenzoic acid (TP-COOH), 4′,((Z,E)-bis(phenylethynylene))dibenzoate (TP-CF_3_-COOCH_3_), and TP-CF_3_-COOH, were designed and synthesized [52,53]. The synthetic details of TP-CF_3_, TP-COOH, and TP-CF_3_-COOCH_3_ are provided in Supplementary Materials (Experimental Section). Synthesis of TP-CF_3_-COOH: The synthetic route is shown in Scheme S1. Add 1.3 g (2.12 mmol) of TP-CF_3_-COOCH_3_ to 30 mL of THF solution and stir, followed by 30 mL of aqueous NaOH solution (25 mmol). The solution was heated to 70 °C until it became turbid and clarified, and then refluxed with heating and stirring. After 24 h of reaction, distill under reduced pressure, add a sufficient amount of 10% dilute hydrochloric acid solution, and precipitate out as the crude product. The crude product obtained by pressure extraction and filtration is recrystallized with n-hexane. The infrared and nuclear magnetic resonance spectroscopy (NMR) characterization (Bruker, Mannheim, Germany) results (Figures S1–S4) confirm the TPB derivatives’ successful synthesis.

### 3.3. Preparation of Probe and Sample Solution

Then, 1 mg of TP-CF_3_-COOH was added to 2 mL of THF, and, after complete solid dissolution, 8 mL of ultra-pure water was added. Then, 1 × 10^5^ μg/L fluorescent probe solution was obtained through ultrasound at room temperature for 30 min. A mixture of THF/water (2:8, *v*/*v*) was used to dilute the 1 × 10^5^ μg/L fluorescent probe solution to the desired working concentration (1 × 10^4^ μg/L) for each use.

### 3.4. Isothermal Titration Calorimetry (ITC)

Due to limitations in instrument corrosion resistance, fw consisting of 80% water and 20% methanol was selected as the mixture for isothermal titration experiments. All experiments were performed at 298 K using the Waters TA Nano-ITC instrument (Waters, Milford, MA, USA). All solutions, including blank and sample solutions, were subjected to ultrasonic treatment for 30 min, and then degassed using a vacuum pump for 15 min. Fentanyl (0.0149 mol)/norfentanyl (0.0215 mol) was added to a mixture of methanol/water (1:4, *v*/*v*) solution and transferred to the sample pool (500 μL). TP-CF_3_-COOH (0.086 mol) was dissolved in methanol/water (1:4, *v*/*v*) solution, and then transferred to a syringe (50 μL). The reference cell (500 μL) was also filled with a degassed methanol/water (1:4, *v*/*v*) solution. The mixing speed was set at 350 rpm. The titration was performed using a 150 s injection interval and a 2.5 μL injection volume. The thermodynamic curve of each binding process is calculated by integrating all peaks, excluding the first one. Each titration experiment was performed in triplicate.

Detailed operational procedures for other methodological experiments, including methods “Visual fluorescence tracking”, “Theoretical calculations”, “Standard curve and methodological validation” and “Rapid qualitative detection of fentanyl”, are provided in Supplementary Materials.

## 4. Conclusions

This is the first instance where we propose that TPB derivatives can be used for the specific detection of fentanyl and norfentanyl, in addition to their mechanoluminescence properties. Our investigation also examines the influence of various chemical groups on the efficiency of fluorescent probes, providing novel insights for the specific detection of fentanyl and norfentanyl in AIE materials. Additionally, this study dynamically monitors the interaction process between probes and fentanyl/norfentanyl, noting changes in the mesoscopic morphology. Finally, this study ultimately elucidates that the fluorescence quenching mechanism between the TP-CF_3_-COOH fluorescent probe and fentanyl/norfentanyl is governed by specific triple-synergy recognition. This recognition facilitates the formation of a unique three-dimensional spatial assembly and molecular conformational changes, thereby enabling fluorescence quenching. Additionally, TSCT occurs during this process, leading to a blue shift in the emission wavelength. Consequently, our fluorescent probe effectively enables the “on–off” visualization and serves as a valuable tool for the onsite screening of fentanyl/norfentanyl. These findings encourage the further exploration of this detection mechanism with other TPB derivatives and suggest its significant potential in identifying unidentified fentanyl analogues.

## Data Availability

The original contributions presented in the study are included in the article; further inquiries can be directed to the corresponding author.

## Supplementary Materials

The following supporting information can be downloaded at: https://www.mdpi.com/article/10.3390/molecules30091985/s1, Scheme S1. The synthetic route of TP-CF_3_-COOH; Scheme S2. The molecular structures of fentanyl and norfentanyl. The molecular structures of fentanyl and norfentanyl. Supplementary Figures: Figure S1. Structural analysis of TP-CF_3_:IR, ^1^H, ^13^C and ^19^F of NMR and HRMS. Figure S2. Structural analysis of TP-CF_3_-COOCH_3_:IR, ^1^H, ^13^C and ^19^F of NMR and HRMS. Figure S3. Structural analysis of TP-COOH:IR, ^1^H, ^13^C and ^19^F of NMR and HRMS. Figure S4. Structural analysis of TP-CF_3_-COOH:IR, ^1^H, ^13^C and ^19^F of NMR and HRMS. Figure S5. PL intensity of TPB at different water contents. Figure S6. PL spectra of 5 AIE materials (100 μg/L) in a mixture of THF/water (1:9, *v*/*v*), and separately added fentanyl/norfentanyl (10 μg/L). Figure S7. The optimization of TP-CF_3_-COOH probe, including the selection of the (A) fw in the THF/water mixtures, (B) ultrasonication, (C) resting stability, (D) temperature stability, (E) sera stability and (F) pH tolerance. Figure S8. X-ray diffraction pattern of solid TP-CF_3_-COOH. Figure S9. Thermogravimetric analysis diagram of solid TP-CF_3_-COOH. Figure S10. ITC of TP-CF_3_-COOH into norfentanyl. Figure S11. ITC background. Figure S12. Conformation of TP-CF_3_-COOH after binding norfentanyl. Figure S13. High content imaging experiment: number of fluorescent dots TP-CF_3_-COOH, TP-CF_3_-COOH + fentanyl and TP-CF_3_-COOH + norfentanyl (THF/water, 1:4, *v*/*v*). Figure S14. Norfentanyl anaglogues and illicit drugs to TP-CF_3_-COOH probe (Molecular formula of 5 chemicals in selectivity evaluation procedure). Figure S15. ^1^H NMR of fentanyl/norfentanyl, TP-CF_3_-COOH probe, TP-CF_3_-COOH + fentanyl/norfentanyl (500 MHz, THF-d_8_/D_2_O, 2:8, *v*/*v*). Figure S16. XPS spectra of (A) TP-CF_3_-COOH probe, (B) TP-CF_3_-COOH + fentanyl and (C) TP-CF_3_-COOH + norfentanyl. Figure S17. A detailed examination of the magnified electrostatic potential on the surface of TP-CF_3_-COOH + fentanyl. Figure S18. The surface penetration of the electrostatic potential of TP-CF_3_-COOH + norfentanyl. To the right lies a magnification of the picture on the left. Figure S19. Ultraviolet spectra of norfentanyl, fentanyl, TP-CF_3_-COOH, TP-CF_3_-COOH + norfentanyl and TP-CF_3_-COOH + fentanyl (THF/water, 1:4, *v*/*v*). Figure S20. Computational Chemistry: Interaction force analysis of TP-CF_3_-COOH + norfentanyl, including IGMH, AIM, sign (lambda2) rho, and EDA-FF. Figure S21. Atomic number of TP-CF_3_-COOH + fentanyl/norfentnayl. Figure S22. The atomic contribution value is visualized in a heat map. Figure S23. Hole-electron transfer diagram of TP-CF_3_-COOH + 3-Methylfentanyl. Figure S24. Hole-electron transfer diagram of TP-CF_3_-COOH + ocfentanyl. Figure S25. Fluorescence change of TP-CF_3_-COOH probe with fentanyl/norfentanyl (THF/ silicone oil, 1:4, *v*/*v*). Figure S26. Quantification of norfentanyl in aqueous solution. Figure S27. Detection of fentanyl analogues (Fentanyl, Depropiony fentanyl, 3-Methylthiofentanyl, Carfentanyl, Norsufentanil, norfentanyl, 36 kinds of fentanyl analogues, β-Hydroxy-3-methylfentanyl, Para-fluorofentany, 4-Fluorobutyrfentanyl, Butyrylfentanyl, 4-Fluoro-isobutyrylfentanyl, Ocfentanyl and Acetylfentanyl) by microplate reader. Supplementary Tables: Table S1. Optimal excitation (EX) and emission (EM) of different AIE materials. Table S2. Gaussian 09 calculated the binding free energy. Table S3. The amount of electron transfer of TP-CF_3_-COOH + fentanyl during electron excitation is calculated by IFCT method in Multiwfn. Table S4. Electron transfer contribution of atoms in TP-CF_3_-COOH + fentanyl S_1_, S_2_ and S_3_ process. Table S5. The amount of electron transfer of TP-CF_3_-COOH + norfentanyl during electron excitation is calculated by IFCT method in Multiwfn. Table S6. Electron transfer contribution of atoms in TP-CF_3_-COOH + norfentanyl S1, S2 and S3 process. Table S7. The amount of electron transfer of TP-CF_3_-COOH + 3-methylfentanyl during electron excitation is calculated by IFCT method in Multiwfn. Table S8. The amount of electron transfer of TP-CF_3_-COOH + ocfentanyl during electron excitation is calculated by IFCT method in Multiwfn. Table S9. Determination of norfentanyl (μg/L) using the developed method and HPLC-MS/MS. Table S10. Determination of fentanyl (μg/L) using the developed method and HPLC-MS/MS. Table S11. Comparison of different methods for the determination of fentanyl. Video S1. Video S2.
