# Supplementary material for: An “On–Off” AIE-Based Lock-and-Key Fluorescent Probe System for Detection of Fentanyl/Norfentanyl
Source: Molecules. 2025 Apr 29;30(9):1985. doi: 10.3390/molecules30091985 (PMC12073365; doi:10.3390/molecules30091985)
Supplement: Supplementary file 1 [file molecules-30-01985-s001.zip › Supporting Information.doc]

**Supplementary Information**

An “On–Off” AIE-Based Lock-and-Key Fluorescent Probe System for Detection of Fentanyl/Norfentanyl

Jing Sun ^1, 4^, Junge Zhi ^2^, Li Zhang ^3^, Yan Qi ^4^, Jiefang Sun ^4^, Yushen Jin ^4^, Jie Yin ^4^, Kai Yao ^1, 4,^ * and Bing Shao ^1, 4,^ *

^1^	National Key Laboratory of Veterinary Public Health Security, College of Veterinary Medicine, China Agricultural University, Beijing 100193, China; sunjingwir4sj@163.com

^2^	School of Chemistry and Chemical Engineering, Beijing Institute of Technology, Beijing 102488, China; jungezhi@bit.edu.cn

^3^	College of Science, China Agricultural University, Beijing 100193, China; zhang_li@cau.edu.cn

^4^	Beijing Key Laboratory of Diagnostic and Traceability Technologies for Food Poisoning, Beijing Center for Disease Prevention and Control, Beijing 100013, China; ingridqiyan@163.com (Y.Q.);
sunjf2001@163.com (J.S.); jinyushen2010@126.com (Y.J.); arnold_jay@163.com (J.Y.)

*****	Correspondence: yaokai@bjcdc.org (K.Y.); shaobingch@sina.com (B.S.)

Table of Contents

**1. Supplementary experimental section**	1

*1.1. Materials and characterization*	1

*1.2 Synthesis of TP-COOH*	1

*1.3 Synthesis of TP-CF_3_*	2

*1.4 Synthesis of TP-CF_3_-COOCH_3_*	3

*1.5 Synthesis of TP-CF_3_-COOH*	4

*1.7 Theoretical calculations*	5

*1.8 Standard curve and methodological validation*	5

*1.9 Rapid qualitative detection of fentanyl*	6

*1.10 Statistical analysis*	6

**2. Supplementary scheme**	6

Scheme S1. The synthetic route of TP-CF_3_-COOH.	7

Scheme S2. The molecular structures of fentanyl and norfentanyl .	7

3. Supplementary figures	8

Figure S1. Structural analysis of TP-CF_3_:IR, ^1^H, ^13^C and ^19^F of NMR and HRMS.	9

Figure S2**.** Structural analysis of TP-CF_3_-COOCH_3_:IR, ^1^H, ^13^C and ^19^F of NMR and HRMS.	10

Figure S3**.** Structural analysis of TP-COOH:IR, ^1^H, ^13^C and ^19^F of NMR and HRMS.	11

Figure S4. Structural analysis of TP-CF_3_-COOH:IR, ^1^H, ^13^C and ^19^F of NMR and HRMS.	12

Figure S5. PL intensity of TPB at different water contents.	12

Figure S6. PL spectra of 5 AIE materials (100 μg/L) in a mixture of THF/water (1:9, v/v), and separately added fentanyl/norfentanyl (10 μg/L).	13

Figure S7. The optimization of TP-CF_3_-COOH probe, including the selection of the (A) fw in the THF/water mixtures, (B) ultrasonication, (C) resting stability, (D) temperature stability, (E) sera stability and (F) pH tolerance.	14

Figure S8. X-ray diffraction pattern of solid TP-CF_3_-COOH.	14

Figure S9. Thermogravimetric analysis diagram of solid TP-CF_3_-COOH.	14

Figure S10. ITC of TP-CF_3_-COOH into norfentanyl.	15

Figure S11. ITC background.	16

Figure S12. Conformation of TP-CF_3_-COOH after binding norfentanyl.	16

Figure S13. High content imaging experiment: number of fluorescent dots TP-CF_3_-COOH, TP-CF_3_-COOH+fentanyl and TP-CF_3_-COOH+norfentanyl (THF/water, 1:4, v/v).	16

Figure S14. Norfentanyl anaglogues and illicit drugs to TP-CF_3_-COOH probe (Molecular formula of 5 chemicals in selectivity evaluation procedure).	17

Figure S15. ^1^H NMR of fentanyl/norfentanyl, TP-CF_3_-COOH probe, TP-CF_3_-COOH+fentanyl/norfentanyl (500 MHz, THF-*d*_8_/D_2_O, 2:8, v/v).	17

Figure S16. XPS spectra of (A) TP-CF_3_-COOH probe, (B) TP-CF_3_-COOH+fentanyl and (C) TP-CF_3_-COOH+norfentanyl.	18

Figure S17. A detailed examination of the magnified electrostatic potential on the surface of TP-CF_3_-COOH+fentanyl.	18

Figure S18. The surface penetration of the electrostatic potential of TP-CF_3_-COOH+norfentanyl. To the right lies a magnification of the picture on the left.	19

Figure S19. Ultraviolet spectra of norfentanyl, fentanyl, TP-CF_3_-COOH, TP-CF_3_-COOH+norfentanyl and TP-CF_3_-COOH+fentanyl (THF/water, 1:4, v/v).	19

Figure S20. Computational Chemistry: Interaction force analysis of TP-CF_3_-COOH+norfentanyl, including IGMH, AIM, sign (lambda2) rho, and EDA-FF.	19

Figure S21. Atomic number of TP-CF_3_-COOH+fentanyl/norfentnayl.	20

Figure S22. The atomic contribution value is visualized in a heat map.	20

Figure S23. Hole-electron transfer diagram of TP-CF_3_-COOH+3-Methylfentanyl.	21

Figure S24. Hole-electron transfer diagram of TP-CF_3_-COOH+ocfentanyl.	21

Figure S25. Fluorescence change of TP-CF_3_-COOH probe with fentanyl/norfentanyl (THF/ silicone oil, 1:4, v/v).	21

Figure S26. Quantification of norfentanyl in aqueous solution.	22

Figure S27. Detection of fentanyl analogues (Fentanyl, Depropiony fentanyl, 3-Methylthiofentanyl, Carfentanyl, Norsufentanil, norfentanyl, 36 kinds of fentanyl analogues，β-Hydroxy-3-methylfentanyl, Para-fluorofentany, 4-Fluorobutyrfentanyl, Butyrylfentanyl, 4-Fluoro-isobutyrylfentanyl, Ocfentanyl and Acetylfentanyl) by microplate reader.	22

4. Supplementary tables	23

Table S1. Optimal excitation (EX) and emission (EM) of different AIE materials.	23

Table S2. Gaussian 09 calculated the binding free energy.	23

Table S3. The amount of electron transfer of TP-CF_3_-COOH+fentanyl during electron excitation is calculated by IFCT method in Multiwfn.	24

Table S4. Electron transfer contribution of atoms in TP-CF_3_-COOH+fentanyl S1, S2 and S3 process.	25

Table S5. The amount of electron transfer of TP-CF_3_-COOH+norfentanyl during electron excitation is calculated by IFCT method in Multiwfn.	34

Table S6. Electron transfer contribution of atoms in TP-CF_3_-COOH+norfentanyl S1, S2 and S3 process.	35

Table S7. The amount of electron transfer of TP-CF_3_-COOH+3-methylfentanyl during electron excitation is calculated by IFCT method in Multiwfn.	43

Table S8. The amount of electron transfer of TP-CF_3_-COOH+ocfentanyl during electron excitation is calculated by IFCT method in Multiwfn.	44

Table S9. Determination of norfentanyl (μg/L) using the developed method and HPLC-MS/MS.	45

Table S10. Determination of fentanyl (μg/L) using the developed method and HPLC-MS/MS.	45

Table S11. Comparison of different methods for the determination of fentanyl.	46

**References**	47

**1. Supplementary experimental section**

*1.1. Materials and characterization*

All reagents and materials are analytically pure and used without any further purification. The chemical reagents and solvents used in this study were purchased from Sigma-Aldrich Trading (Shanghai), Shanghai Aladdin Biochemistry Ltd. and Beijing Bailingway Technology Co. In addition, norfentanyl was purchased from Shanghai Shaoyuan Reagent Co. Fentanyl and its analogues standards were purchased from Tianjin Alta (nitrogen blowing was performed to obtain pure products). These standards are stored at -20°C and returned to room temperature before use. H_2_O-*d*_2_, DMSO-*d*_6_ and THF-*d*_8_ were purchased from Qingdao Tenglong Microwave Technology Co. Microporous water purification system (Milli-Q) produces ultrapure water for the entire research process.

According to different experimental requirements, different deuterium solvents were selected, and NMR spectra were obtained by Bruker NEO 600. TEM images were obtained using Hitachi HT7800 transmission electron microscope (Hitachi, Japan). Scanning electron microscope images were obtained by Hitachi S-4800 scanning electron microscope. Ultraviolet-visible-near-infrared absorption spectra were collected using an ultraviolet-visible spectrophotometer. The fluorescence spectra and fluorescence quantum yield of TPB series materials were obtained by using a fluorescence spectrometer with integrating sphere (RF 6000). The fluorescence lifetime was measured by the steady-state transient fluorescence spectrometer (FLS1000/FS5). Thermogravimetric analysis (TGA) results were measured on the Shimadzu TGA-50 thermal analyzer. Take dark field digital photos with your mobile phone (Huawei mate30 pro and iPhone 12). XPS electron spectroscopy (Thermo Field, USA) analysis of XPS spectra in situ.

*1.2 Synthesis of TP-COOH*

To synthesize TP-COOH, a mixture was prepared by adding 10.2 g (100 mmol) of phenylacetylene, 50 g (223.84 mmol) of copper bromide and 1 g (3.76 mmol) of palladium bromide to 100 mL of toluene and 2 mL of acetonitrile mixture solvent. The mixture was stirred at room temperature for 12 h and then filtered under reduced pressure, concentrated and dried by rotary evaporation. To purify the intermediate (designated as compound 1), a silica gel column chromatography was performed with dichloromethane/petroleum ether (1:2, v/v) as the eluents. The mixture of liquid nitrogen freeze degassed solution of toluene (30 mL) and methanol (10 mL) was repeated three times. Next, 4-methoxycarbonylphenylboronic (269.95 mg, 1.50 mmol), yield intermediate 1 (250.00 mg of the above substance, 0.50 mmol), Pd(PPh_3_)_4_ (61.25 mg, 0.05 mmol) and potassium carbonate (414.63 mg, 3 mmol) were added under nitrogen atmosphere. This solution was heated to 80 ℃ and refluxed with stirring for 24 h. After the reaction, the collected filtrate was washed three times with 5% dilute hydrochloric acid solution under reduced pressure to remove the potassium carbonate. Anhydrous magnesium sulfate was added. After filtration, the product was concentrated by distillation under reduced pressure and purified by silica gel column chromatography (dichloromethane/hexane = 1:1, v/v). The resulting solid was recrystallized and filtered, yielding the powder TP-COOCH_3_. Subsequently, 1 g (2.11 mmol) of TP-COOCH_3_ was dissolved in 30 mL of THF, and mixed with 30 mL of sodium hydroxide (NaOH) aqueous (25 mmol). The solution was heated to 70°C until it became turbid and clarified, and then allowed to reflux with stirring. After 24 h of reaction, the mixture was distilled under reduced pressure, followed by addition of 10% dilute hydrochloric acid to precipitate the crude product. The crude product obtained by pressure extraction and filtration is recrystallized with n-hexane. A light blue-green solid powder was obtained (87%). ^1^H NMR (500 MHz, DMSO-*d*_6_) δ spectrum showed (ppm):δ 13.02 (s, 2H), 8.06 (s, 4H), 7.42 (d, J = 8.2 Hz, 4H), 7.29 (d, J = 17.9 Hz, 6H),7.06 (s, 2H), 6.67 (s, 2H). ^13^C NMR (126 MHz, DMSO-*d*_6_) δ 166.95, 143.38, 143.30, 140.96, 130.33, 129.39, 128.47, 127.88, 127.08. MS: [M-H]^-^, m/z, 445.1445 (Calcd. 445.1445).

*1.3 Synthesis of TP-CF_3_*

To synthesize TP-CF_3_, 0.51 g (3 mmol) of 4-ethynyltrifluorotoluene, 0.04 g (0.15 mmol) of palladium bromide, and 2.01 g (9 mmol) of copper bromide were mixed in 25 mL of toluene and 0.5 mL of acetonitrile, then stirred for 12 h at room temperature and shielded from light. The mixture was then filtered under reduced pressure, concentrate and dried using rotary evaporation. The resulting TPB-CF_3_-Br was purified by silica gel column chromatography using dichloromethane/petroleum ether (1:2, v/v) as the eluents. The freeze-degassed solution of toluene (30 mL) and methanol (10 mL) was treated in liquid nitrogen and repeated three times. Nextly, phenylboronic acid (182.89 mg, 1.50 mmol), TPB-CF_3_-Br (250.00 mg, 0.50 mmol), Pd(PPh_3_)_4_ (61.25 mg, 0.05 mmol) and potassium carbonate (414.63 mg, 3 mmol) were added under nitrogen protection. The mixture was heated to 80 °C and refluxed with stirring for 24 h. After the reaction, the filtrate was washed three times with 5% dilute hydrochloric acid solution under reduced pressure to remove the potassium carbonate. Anhydrous magnesium sulfate was added to dry the productThe resulting solid was recrystallized and filtered to yield TP-CF3. The ^1^H NMR (500 MHz, DMSO-*d*_6_) spectrum showed δ (ppm): 7.98 (d, J = 8.2 Hz, 2H), 7.91 – 7.82 (m, 4H), 7.66 (d, J = 8.5 Hz, 2H), 7.56 – 7.48 (m, 4H), 7.33 – 7.25 (m, 2H), 7.12 – 7.05 (m, 1H), 6.75 – 6.64 (m, 1H). After filtration, the product was concentrated by distillation under reduced pressure and separated by silica gel column chromatography with dichloromethane/hexane (1:1, v/v) as eluent, and the resulting solid was recrystallized and filtered to obtain the powder TP-CF_3_. A light yellow solid powder was obtained (47%). ^1^H NMR (500 MHz, DMSO-*d*_6_) spectrum showed δ (ppm):δ 7.86 (d, J = 8.2 Hz, 4H), 7.65 (m, 4H), 7.5 (d, J = 8.5 Hz, 4H), 7.3 (m, 4H), 7.02(m, 2H), 6.69 (m, 2H). ^13^C NMR (126 MHz, DMSO-*d*_6_) δ 145.15, 143.28, 142.84, 137.85, 130.82, 128.42, 128.35, 127.90, 127.56, 126.96, 125.65, 125.15, 67.06. ^19^F NMR (470 MHz, DMSO-*d*_6_) δ -61.03. MS: [M-H]^-^, m/z, 493.1396 (Calcd. 493.1391).

*1.4 Synthesis of TP-CF_3_-COOCH_3_*

4-trifluoromethylphenylboronic acid (302 mg, 1.59 mmol), yield intermediate 1 (255.70 mg of the above substance, 0.53 mmol), Pd(PPh_3_)_4_ (61.25 mg, 0.05 mmol) and potassium carbonate (439.5 mg, 3.18 mmol) were added to freeze-degassed solution of toluene (30 mL) and methanol (10 mL) under nitrogen atmosphere, heated to 80 ℃ and stirred at refluxing for 24 h. After the reaction, the collected filtrate was washed three times with 5% dilute hydrochloric acid solution under reduced pressure to remove the potassium carbonate, after which anhydrous magnesium sulfate was added to remove water and dried. After filtration, the product was concentrated by distillation under reduced pressure and separated by silica gel column chromatography (dichloromethane/hexane = 1:1, v/v), and the resulting solid was recrystallized and filtered to obtain the powder TP-CF_3_-COOCH_3_. A yellow solid powder was obtained (83%). ^1^H NMR (500 MHz, DMSO-*d*_6_) spectrum showed δ (ppm): δ 8.09 (d, J = 8.2 Hz, 2H), 7.88 (s, 2H), 7.55 (s, 2H), 7.33 (d, J = 8.2 Hz, 2H), 6.77 (dd, J = 11.5, 7.9 Hz, 2H), 3.89 (s, 6H). ^13^C NMR (126 MHz, DMSO-*d*_6_) δ 165.75, 142.61, 140.28, 139.25, 132.08, 130.98, 129.75, 129.65, 129.62, 127.81, 125.83, 52.68, 0.27. ^19^F NMR (470 MHz, DMSO-*d*_6_) δ -60.96.MS: [M-H]^-^, m/z, 609.1506 (Calcd. 609.1516).

*1.5 Synthesis of TP-CF_3_-COOH*

The synthetic route is shown in Scheme S1. Add 1.3 g (2.12 mmol) of TP-CF_3_-COOCH_3_ to 30 mL of THF solution and stir, followed by 30 mL of aqueous NaOH solution (25 mmol). The solution was heated to 70°C until it became turbid and clarified, and then refluxed with heating and stirring. After 24 h of reaction, distill under reduced pressure, add a sufficient amount of 10% dilute hydrochloric acid solution and precipitate out as the crude product. The crude product obtained by pressure extraction and filtration is recrystallized with n-hexane. A yellowish-green solid powder was obtained (95%). ^1^H NMR (500 MHz, DMSO-*d*_6_) δ (ppm): δ 12.98 (s, 2H), 7.87 (dd, J = 8.4, 2.6 Hz, 8H), 7.55 (d, J = 7.9 Hz, 4H), 7.22 (d, J = 8.3 Hz, 4H), 6.75 (s, 2H). ^13^C NMR (126 MHz, DMSO-*d*_6_) δ 166.69, 144.77, 143.17, 142.45, 131.08, 130.07, 129.54, 127.39, 126.92, 125.50. ^19^F NMR (470 MHz, DMSO-*d*_6_) δ -60.98. MS: [M-H]^-^, m/z, 581.1193 (Calcd. 581.1182).

*1.6 Visual fluorescence tracking*

20 μL of fentanyl solution (1000 μg/L) were added into the 96-well plate containing 180 μL of 1 × 10^4^ μg/L ultrasonic probe solution. Then the 96-well plate was then immediately placed in a HCI instrument and filmed the entire fluorescence dimming process, as well as recording fluorescence images and videos at different time points. Compared with the control group with pure solvent, the fluorescence change process after contact between the fluorescence probe and the object to be measured was captured, and the real size of the graphic pixel was used as the scale reference.

*1.7 Theoretical calculations*

In order to study the interaction between fentanyl/norfentanyl molecules and TP-CF_3_-COOH, an optimization strategy combining global semi-empirical quantum mechanical search and DFT was employed to identify the most energy stable self-organization structure from more than 350 possible conformation. Molecular docking is first performed using MOE (ULC, 2020) software, with a force field of MMFF94x and a scoring function of GBVI/WSA dG. The lowest energy geometry was selected for subsequent DFT optimization and interaction energy. Gaussian 09 software was utilized for subsequent DFT and TDDFT calculations, with OPT-FREQ and B3LYP/6-31G* selected as conformational optimization and foundation set, respectively. The optimization and convergence energies of fentanyl alone, norfentanyl, and TP-CF_3_-COOH were calculated. In addition, the correction energy of the conformation of the two self-organizing bodies is calculated. In the calculation of TDDFT, CAM-B3LYP-d3 is used as the base set of the above optimized conformation, and a water solvent model was selected.

The vertical excitation energy of each component's S_1_, S_2_, and S_3_ transitions is calculated, and Multiwfn analyzed all wave function results. Based on the output of Multiwfn, the VMD program drew all isosurface plots.

*1.8 Standard curve and methodological validation*

The fentanyl and norfentanyl standard solutions were individually prepared in ultra-pure water and fetal bovine serum, respectively, spanning concentrations from 0.01 to 1000 μg/L. Tetrahydrofuran was utilized to effectively precipitate the proteins during the sample cleanup process. To ascertain linearity, the standard solution (20 μL) was incubated with the TP-CF_3_-COOH fluorescent probe (180 μL, 1 × 10^4^ μg/L) at 25℃ for 30 s. Subsequently, the fluorescence intensity of the resultant mixture was measured using a portable microplate reader with an excitation wavelength (λ_ex_) of 350 nm and a slit width of 20 nm. Additionally, spiked samples were prepared with concentrations of 1000, 100 and 1 μg/L as high, medium and low controls, respectively. These samples were used to systematically evaluate and compare the sensitivity and accuracy of the instrument method and our probe detection method.

*1.9 Rapid qualitative detection of fentanyl*

In Scheme 1, a solution containing 20 uL of fentanyl at a concentration of 50 μg/L was added to 180 μL of 1 × 10^4^ μg/L TP-CF_3_-COOH fluorescent probe solution. Upon exposure to UV irradiation for 60 s, the fluorescence intensity of the solution was significantly quenched, enabling rapid qualitative detection of fentanyl.

*1.10 Statistical analysis*

Statistical analysis was performed using Origin version 8.0. The significance levels were denoted by asterisks, where * indicates *p* <0.05, ** indicates *p* <0.01, and *** indicates *p* <0.001, all of which were considered statistically significant.

**2. Supplementary scheme**

Scheme S1. The synthetic route of TP-CF_3_-COOH.

Scheme S2. The molecular structures of fentanyl and norfentanyl .

3. Supplementary figures

Figure S1. Structural analysis of TP-CF_3_:IR, ^1^H, ^13^C and ^19^F of NMR and HRMS.

Figure S2**.** Structural analysis of TP-CF_3_-COOCH_3_:IR, ^1^H, ^13^C and ^19^F of NMR and HRMS.

Figure S3**.** Structural analysis of TP-COOH:IR, ^1^H, ^13^C and ^19^F of NMR and HRMS.

Figure S4. Structural analysis of TP-CF_3_-COOH:IR, ^1^H, ^13^C and ^19^F of NMR and HRMS.

Figure S5. PL intensity of TPB at different water contents.

Figure S6. PL spectra of 5 AIE materials (100 μg/L) in a mixture of THF/water (1:9, v/v), and separately added fentanyl/norfentanyl (10 μg/L).

Figure S7. The optimization of TP-CF_3_-COOH probe, including the selection of the (A) fw in the THF/water mixtures, (B) ultrasonication, (C) resting stability, (D) temperature stability, (E) sera stability and (F) pH tolerance.

Figure S8. X-ray diffraction pattern of solid TP-CF_3_-COOH.

Figure S9. Thermogravimetric analysis diagram of solid TP-CF_3_-COOH.

Figure S10. ITC of TP-CF_3_-COOH into norfentanyl.

Figure S11. ITC background.

Figure S12. Conformation of TP-CF_3_-COOH after binding norfentanyl.

Figure S13. High content imaging experiment: number of fluorescent dots TP-CF_3_-COOH, TP-CF_3_-COOH+fentanyl and TP-CF_3_-COOH+norfentanyl (THF/water, 1:4, v/v).

Figure S14. Norfentanyl anaglogues and illicit drugs to TP-CF_3_-COOH probe (Molecular formula of 5 chemicals in selectivity evaluation procedure).

Figure S15. ^1^H NMR of fentanyl/norfentanyl, TP-CF_3_-COOH probe, TP-CF_3_-COOH+fentanyl/norfentanyl (500 MHz, THF-*d*_8_/D_2_O, 2:8, v/v).

Figure S16. XPS spectra of (A) TP-CF_3_-COOH probe, (B) TP-CF_3_-COOH+fentanyl and (C) TP-CF_3_-COOH+norfentanyl.

Figure S17. A detailed examination of the magnified electrostatic potential on the surface of TP-CF_3_-COOH+fentanyl.

Figure S18. The surface penetration of the electrostatic potential of TP-CF_3_-COOH+norfentanyl. To the right lies a magnification of the picture on the left.

Figure S19. Ultraviolet spectra of norfentanyl, fentanyl, TP-CF_3_-COOH, TP-CF_3_-COOH+norfentanyl and TP-CF_3_-COOH+fentanyl (THF/water, 1:4, v/v).

Figure S20. Computational Chemistry: Interaction force analysis of TP-CF_3_-COOH+norfentanyl, including IGMH, AIM, sign (lambda2) rho, and EDA-FF.

Figure S21. Atomic number of TP-CF_3_-COOH+fentanyl/norfentnayl.

Figure S22. The atomic contribution value is visualized in a heat map.

Figure S23. Hole-electron transfer diagram of TP-CF_3_-COOH+3-Methylfentanyl.

Figure S24. Hole-electron transfer diagram of TP-CF_3_-COOH+ocfentanyl.

Figure S25. Fluorescence change of TP-CF_3_-COOH probe with fentanyl/norfentanyl (THF/ silicone oil, 1:4, v/v).

Figure S26. Quantification of norfentanyl in aqueous solution.

Figure S27. Detection of fentanyl analogues (Fentanyl, Depropiony fentanyl, 3-Methylthiofentanyl, Carfentanyl, Norsufentanil, norfentanyl, 36 kinds of fentanyl analogues，β-Hydroxy-3-methylfentanyl, Para-fluorofentany, 4-Fluorobutyrfentanyl, Butyrylfentanyl, 4-Fluoro-isobutyrylfentanyl, Ocfentanyl and Acetylfentanyl) by microplate reader.

4. Supplementary tables

| nm | TPB | TP-CF_3_ | TP-COOH | TP-CF_3_-COOCH_3_ | TP-CF_3_-COOH |
| --- | --- | --- | --- | --- | --- |
| EX | 253 | 310 | 370 | 354 | 360 |
| EM | 388 | 408 | 433 | 461 | 470\ |

Table S1. Optimal excitation (EX) and emission (EM) of different AIE materials.

Table S2. Gaussian 09 calculated the binding free energy.

| Complexes | Weak interaction | Binding energy (kJ/mol) |
| --- | --- | --- |
| TPB+fentanyl | H-π | -13.85 |
| TPB+norfentanyl | H-π | -14.19 |
| TP-COOH+fentanyl | H-π | -14.78 |
| TP-COOH+norfentanyl | H-π、π-π | -14.93 |
| TP-CF_3_+fentanyl | H-π、H-F | -14.03 |
| TP-CF_3_+norfentanyl | H-π、H-F | -14.95 |
| TP-CF_3_-COOCH_3_+fentanyl | H-π、H-F、π-π | -14.86 |
| TP-CF_3_-COOCH_3_+norfentanyl | H-π、H-F、π-π | -13.39 |
| TP-CF_3_-COOH+fentanyl | H-π、π-π、H-F | **-15.41** |
| TP-CF_3_-COOH+norfentanyl | H-π、H-F、π-π | **-15.08** |
| TPB+Cocaine | / | -0.11 |
| TPB+Heroin | / | 0.18 |
| TP-CF_3_-COOH+Cocaine | / | -0.25 |
| TP-CF_3_-COOH+Heroin | / | 0.23 |

Table S3. The amount of electron transfer of TP-CF_3_-COOH+fentanyl during electron excitation is calculated by IFCT method in Multiwfn.

| Excited  state | Fragment | Atom   indices | Each fragment  to hole | Each fragment   to electron | Transferred electrons  between fragments |
| --- | --- | --- | --- | --- | --- |
|  | 1 | 1-53 | -0.03% | 0.23% | 1 -> 2: 0.00000 |
| S1 (LE) |  |  |  |  | 1 <- 2: 0.00230 |
|  | 2 | 54-115 | 100.03% | 99.77% | Net 1 -> 2: -0.00230 |
|  | 1 | 1-53 | 99.00% | 0.6% | 1 -> 2: 0.98446 |
| S2 (IC) |  |  |  |  | 1 <- 2: 0.00006 |
|  | 2 | 54-115 | 1.00% | 99.4% | Net 1 -> 2: 0.98440 |
|  | 1 | 1-53 | -0.13% | 0.43% | 1 -> 2: 0.00000 |
| S3 (LE) |  |  |  |  | 1 <- 2: 0.00431 |
|  | 2 | 54-115 | 100.13% | 99.57% | Net 1 -> 2:-0.00431 |

Table S4. Electron transfer contribution of atoms in TP-CF_3_-COOH+fentanyl S1, S2 and S3 process.

| Excited state | Heat map serial number | Atomic number | Hole | Electron | Overlap | Diff. |
| --- | --- | --- | --- | --- | --- | --- |
| S1 | 1 | 1 (C) | 0.00% | 0.00% | 0.00% | 0.00% |
|  | 2 | 2 (C) | 0.00% | 0.00% | 0.00% | 0.00% |
|  | 3 | 3 (C) | 0.00% | 0.00% | 0.00% | 0.00% |
|  | 4 | 4 (O) | 0.01% | 0.00% | 0.00% | -0.01% |
|  | 5 | 5 (N) | 0.00% | 0.00% | 0.00% | 0.00% |
|  | 6 | 6 (C) | 0.00% | 0.00% | 0.00% | 0.00% |
|  | 7 | 7 (C) | -0.01% | 0.00% | 0.00% | 0.01% |
|  | 8 | 8 (C) | 0.00% | 0.01% | 0.00% | 0.01% |
|  | 9 | 9 (N) | -0.05% | 0.00% | 0.00% | 0.05% |
|  | 10 | 10 (C) | 0.00% | 0.00% | 0.00% | 0.00% |
|  | 11 | 11 (C) | 0.00% | 0.00% | 0.00% | 0.00% |
|  | 12 | 12 (C) | 0.00% | 0.00% | 0.00% | 0.00% |
|  | 13 | 13 (C) | 0.00% | 0.00% | 0.00% | 0.00% |
|  | 14 | 14 (C) | 0.00% | 0.00% | 0.00% | 0.00% |
|  | 15 | 15 (C) | 0.00% | 0.00% | 0.00% | 0.00% |
|  | 16 | 16 (C) | 0.00% | 0.00% | 0.00% | 0.00% |
|  | 17 | 17 (C) | 0.00% | 0.00% | 0.00% | 0.00% |
|  | 18 | 18 (C) | 0.01% | 0.01% | 0.01% | 0.00% |
|  | 19 | 19 (C) | 0.00% | 0.00% | 0.00% | 0.00% |
|  | 20 | 20 (C) | 0.00% | 0.00% | 0.00% | 0.00% |
|  | 21 | 21 (C) | 0.00% | 0.00% | 0.00% | 0.00% |
|  | 22 | 22 (C) | 0.00% | 0.01% | 0.00% | 0.02% |
|  | 23 | 23 (C) | 0.00% | 0.00% | 0.00% | 0.00% |
|  | 24 | 24 (C) | 0.00% | 0.00% | 0.00% | 0.01% |
|  | 25 | 25 (C) | 0.00% | 0.00% | 0.00% | 0.01% |
|  | 26 | 54 (C) | 5.08% | 4.94% | 5.01% | -0.14% |

Table S4 (continued). Electron transfer contribution of atoms in TP-CF_3_-COOH+fentanyl S1, S2 and S3 process.

| Excited state | Heat map serial number | Atomic number | Hole | Electron | Overlap | Diff. |
| --- | --- | --- | --- | --- | --- | --- |
| S1 | 27 | 55 (C) | 0.23% | 1.80% | 0.65% | 1.57% |
|  | 28 | 56 (C) | 4.11% | 2.84% | 3.42% | -1.27% |
|  | 29 | 57 (C) | 2.95% | 4.72% | 3.73% | 1.77% |
|  | 30 | 58 (C) | 3.88% | 3.90% | 3.89% | 0.02% |
|  | 31 | 59 (C) | 0.25% | 0.98% | 0.49% | 0.74% |
|  | 32 | 60 (C) | 15.87% | 12.89% | 14.30% | -2.98% |
|  | 33 | 61 (C) | 11.46% | 10.19% | 10.81% | -1.28% |
|  | 34 | 62 (C) | 10.65% | 9.37% | 9.99% | -1.28% |
|  | 35 | 63 (C) | 16.02% | 13.03% | 14.45% | -2.99% |
|  | 36 | 64 (C) | 1.07% | 0.67% | 0.84% | -0.40% |
|  | 37 | 65 (C) | 2.68% | 4.51% | 3.48% | 1.83% |
|  | 38 | 66 (C) | 0.89% | 0.57% | 0.71% | -0.32% |
|  | 39 | 67 (C) | 1.64% | 0.91% | 1.22% | -0.73% |
|  | 40 | 68 (C) | 0.11% | 0.42% | 0.22% | 0.30% |
|  | 41 | 69 (C) | 1.52% | 1.22% | 1.37% | -0.30% |
|  | 42 | 70 (C) | 0.12% | 0.10% | 0.11% | -0.02% |
|  | 43 | 71 (C) | 1.62% | 1.34% | 1.47% | -0.28% |
|  | 44 | 72 (C) | 3.65% | 3.54% | 3.59% | -0.11% |
|  | 45 | 73 (C) | 0.26% | 1.00% | 0.51% | 0.74% |
|  | 46 | 74 (C) | 4.65% | 4.70% | 4.68% | 0.05% |
|  | 47 | 75 (C) | 0.21% | 1.62% | 0.58% | 1.41% |
|  | 48 | 76 (C) | 3.87% | 2.94% | 3.37% | -0.94% |
|  | 49 | 77 (C) | 1.45% | 0.75% | 1.04% | -0.70% |
|  | 50 | 78 (C) | 0.10% | 0.44% | 0.21% | 0.34% |
|  | 51 | 79 (C) | 1.23% | 0.99% | 1.10% | -0.25% |

Table S4 (continued). Electron transfer contribution of atoms in TP-CF_3_-COOH+fentanyl S1, S2 and S3 process.

| Excited state | Heat map serial number | Atomic number | Hole | Electron | Overlap | Diff. |
| --- | --- | --- | --- | --- | --- | --- |
|  | 52 | 80 (C) | 0.11% | 0.08% | 0.10% | -0.03% |
|  | 53 | 81 (C) | 1.41% | 1.15% | 1.27% | -0.25% |
|  | 54 | 82 (C) | 0.19% | 1.81% | 0.59% | 1.62% |
|  | 55 | 83 (O) | 0.80% | 1.53% | 1.11% | 0.73% |
|  | 56 | 84 (O) | 0.17% | 0.47% | 0.29% | 0.30% |
|  | 57 | 85 (C) | 0.18% | 1.69% | 0.55% | 1.51% |
|  | 58 | 86 (O) | 0.71% | 1.44% | 1.01% | 0.73% |
| S1 | 59 | 87 (O) | 0.17% | 0.44% | 0.27% | 0.27% |
|  | 60 | 88 (C) | 0.08% | 0.13% | 0.10% | 0.04% |
|  | 61 | 89 (F) | 0.05% | 0.04% | 0.05% | -0.01% |
|  | 62 | 90 (F) | 0.02% | 0.02% | 0.02% | 0.00% |
|  | 63 | 91 (F) | 0.02% | 0.02% | 0.02% | 0.00% |
|  | 64 | 92 (C) | 0.07% | 0.11% | 0.09% | 0.04% |
|  | 65 | 93 (F) | 0.04% | 0.04% | 0.04% | 0.00% |
|  | 66 | 94 (F) | 0.03% | 0.02% | 0.02% | -0.01% |
|  | 67 | 95 (F) | 0.01% | 0.01% | 0.01% | 0.00% |
|  | Sum of hole shown above | | 99.61% | Sum of electron shown above | | 99.43% |
|  | 1 | 1 (C) | 0.00% | 0.00% | 0.00% | 0.00% |
|  | 2 | 2 (C) | 0.01% | 0.00% | 0.00% | -0.01% |
|  | 3 | 3 (C) | 0.10% | 0.00% | 0.01% | -0.10% |
| S2 | 4 | 4 (O) | 0.08% | 0.00% | 0.01% | -0.08% |
|  | 5 | 5 (N) | 0.31% | 0.00% | 0.02% | -0.30% |
|  | 6 | 6 (C) | 0.54% | 0.00% | 0.00% | -0.54% |
|  | 7 | 7 (C) | 6.47% | 0.01% | 0.24% | -6.46% |

Table S4 (continued). Electron transfer contribution of atoms in TP-CF_3_-COOH+fentanyl S1, S2 and S3 process.

| Excited state | Heat map serial number | Atomic number | Hole | Electron | Overlap | Diff. |
| --- | --- | --- | --- | --- | --- | --- |
| S2 | 8 | 8 (C) | 3.00% | 0.02% | 0.24% | -2.98% |
|  | 9 | 9 (N) | 63.01% | 0.01% | 0.64% | -63.00% |
|  | 10 | 10 (C) | 2.34% | 0.01% | 0.15% | -2.33% |
|  | 11 | 11 (C) | 0.57% | 0.00% | 0.00% | -0.57% |
|  | 12 | 12 (C) | 0.60% | 0.00% | 0.04% | -0.59% |
|  | 13 | 13 (C) | 0.14% | 0.00% | 0.00% | -0.14% |
|  | 14 | 14 (C) | 0.09% | 0.00% | 0.01% | -0.09% |
|  | 15 | 15 (C) | 0.20% | 0.01% | 0.03% | -0.19% |
|  | 16 | 16 (C) | 0.00% | 0.01% | 0.00% | 0.01% |
|  | 17 | 17 (C) | 0.22% | 0.00% | 0.00% | -0.22% |
|  | 18 | 18 (C) | 2.68% | 0.04% | 0.31% | -2.65% |
|  | 19 | 19 (C) | 5.96% | 0.00% | 0.09% | -5.95% |
|  | 20 | 20 (C) | 0.20% | 0.00% | 0.02% | -0.20% |
|  | 21 | 21 (C) | 0.14% | 0.01% | 0.03% | -0.13% |
|  | 22 | 22 (C) | 0.01% | 0.02% | 0.01% | 0.01% |
|  | 23 | 23 (C) | 0.06% | 0.00% | 0.00% | -0.06% |
|  | 24 | 24 (C) | 0.04% | 0.00% | 0.01% | -0.04% |
|  | 25 | 25 (C) | 0.02% | 0.00% | 0.00% | -0.02% |
|  | 26 | 54 (C) | 0.05% | 10.59% | 0.70% | 10.54% |
|  | 22 | 22 (C) | 0.01% | 0.02% | 0.01% | 0.01% |
|  | 23 | 23 (C) | 0.06% | 0.00% | 0.00% | -0.06% |
|  | 24 | 24 (C) | 0.04% | 0.00% | 0.01% | -0.04% |
|  | 25 | 25 (C) | 0.02% | 0.00% | 0.00% | -0.02% |
|  | 26 | 54 (C) | 0.05% | 10.59% | 0.70% | 10.54% |
|  | 27 | 55 (C) | 0.06% | 5.34% | 0.59% | 5.27% |

Table S4 (continued). Electron transfer contribution of atoms in TP-CF_3_-COOH+fentanyl S1, S2 and S3 process.

| Excited state | Heat map serial number | Atomic number | Hole | Electron | Overlap | Diff. |
| --- | --- | --- | --- | --- | --- | --- |
| S2 | 28 | 56 (C) | 0.11% | 3.60% | 0.63% | 3.49% |
|  | 29 | 57 (C) | 0.01% | 10.60% | 0.24% | 10.60% |
|  | 30 | 58 (C) | 0.10% | 4.31% | 0.67% | 4.21% |
|  | 31 | 59 (C) | 0.29% | 4.48% | 1.14% | 4.19% |
|  | 32 | 60 (C) | -0.01% | 8.19% | 0.00% | 8.20% |
|  | 33 | 61 (C) | -0.01% | 10.25% | 0.00% | 10.26% |
|  | 34 | 62 (C) | -0.01% | 4.26% | 0.00% | 4.27% |
|  | 35 | 63 (C) | -0.01% | 8.95% | 0.00% | 8.96% |
|  | 36 | 64 (C) | 0.00% | 0.23% | 0.02% | 0.22% |
|  | 37 | 65 (C) | 0.00% | 1.42% | 0.00% | 1.42% |
|  | 38 | 66 (C) | 0.00% | 0.36% | 0.00% | 0.36% |
|  | 39 | 67 (C) | 0.00% | 0.41% | 0.00% | 0.41% |
|  | 40 | 68 (C) | 0.00% | 0.18% | 0.01% | 0.18% |
|  | 41 | 69 (C) | 0.00% | 0.42% | 0.00% | 0.42% |
|  | 42 | 70 (C) | 0.00% | 0.03% | 0.00% | 0.03% |
|  | 43 | 71 (C) | 0.00% | 0.70% | 0.00% | 0.70% |
|  | 44 | 72 (C) | 0.00% | 1.59% | 0.00% | 1.59% |
|  | 45 | 73 (C) | 0.00% | 0.38% | 0.00% | 0.38% |
|  | 46 | 74 (C) | 0.00% | 2.05% | 0.00% | 2.05% |
|  | 47 | 75 (C) | 0.00% | 0.53% | 0.01% | 0.53% |
|  | 48 | 76 (C) | 0.00% | 1.49% | 0.00% | 1.49% |
|  | 49 | 77 (C) | 0.01% | 0.47% | 0.05% | 0.46% |
|  | 50 | 78 (C) | 0.01% | 0.44% | 0.07% | 0.43% |
|  | 51 | 79 (C) | 0.00% | 0.85% | 0.06% | 0.85% |
|  | 52 | 80 (C) | 0.00% | 0.06% | 0.01% | 0.06% |

Table S4 (continued). Electron transfer contribution of atoms in TP-CF_3_-COOH+fentanyl S1, S2 and S3 process.

| Excited state | Heat map serial number | Atomic number | Hole | Electron | Overlap | Diff. |
| --- | --- | --- | --- | --- | --- | --- |
|  | 53 | 81 (C) | 0.01% | 0.84% | 0.07% | 0.84% |
|  | 54 | 82 (C) | 0.01% | 7.61% | 0.29% | 7.60% |
|  | 55 | 83 (O) | 0.02% | 5.21% | 0.35% | 5.19% |
|  | 56 | 84 (O) | 0.00% | 1.68% | 0.00% | 1.68% |
|  | 57 | 85 (C) | 0.00% | 0.59% | 0.00% | 0.59% |
|  | 58 | 86 (O) | 0.00% | 0.55% | 0.00% | 0.55% |
|  | 59 | 87 (O) | 0.00% | 0.17% | 0.01% | 0.17% |
| S2 | 60 | 88 (C) | 0.00% | 0.04% | 0.00% | 0.04% |
|  | 61 | 89 (F) | 0.00% | 0.01% | 0.00% | 0.01% |
|  | 62 | 90 (F) | 0.00% | 0.01% | 0.00% | 0.01% |
|  | 63 | 91 (F) | 0.00% | 0.01% | 0.00% | 0.01% |
|  | 64 | 92 (C) | 0.00% | 0.13% | 0.00% | 0.13% |
|  | 65 | 93 (F) | 0.00% | 0.04% | 0.01% | 0.04% |
|  | 66 | 94 (F) | 0.00% | 0.02% | 0.00% | 0.02% |
|  | 67 | 95 (F) | 0.00% | 0.01% | 0.01% | 0.01% |
|  | Sum of hole shown above | | 87.43% | Sum of electron shown above | | 99.22% |
|  | 1 | 1 (C) | 0.00% | 0.00% | 0.00% | 0.00% |
|  | 2 | 2 (C) | 0.00% | 0.00% | 0.00% | 0.00% |
|  | 3 | 3 (C) | 0.00% | 0.00% | 0.01% | 0.00% |
| S3 | 4 | 4 (O) | -0.03% | 0.00% | 0.01% | 0.04% |
|  | 5 | 5 (N) | -0.05% | 0.00% | 0.00% | 0.05% |
|  | 6 | 6 (C) | 0.00% | 0.00% | 0.00% | 0.00% |
|  | 7 | 7 (C) | -0.01% | 0.01% | 0.00% | 0.01% |
|  | 8 | 8 (C) | 0.00% | 0.01% | 0.00% | 0.01% |

Table S4 (continued). Electron transfer contribution of atoms in TP-CF_3_-COOH+fentanyl S1, S2 and S3 process.

| Excited state | Heat map serial number | Atomic number | Hole | Electron | Overlap | Diff. |
| --- | --- | --- | --- | --- | --- | --- |
| S3 | 9 | 9 (N) | -0.06% | 0.01% | 0.00% | 0.06% |
|  | 10 | 10 (C) | 0.00% | 0.00% | 0.00% | 0.01% |
|  | 11 | 11 (C) | 0.00% | 0.00% | 0.00% | 0.00% |
|  | 12 | 12 (C) | -0.01% | 0.00% | 0.00% | 0.01% |
|  | 13 | 13 (C) | -0.02% | 0.00% | 0.00% | 0.02% |
|  | 14 | 14 (C) | 0.00% | 0.00% | 0.01% | 0.01% |
|  | 15 | 15 (C) | 0.00% | 0.01% | 0.00% | 0.01% |
|  | 16 | 16 (C) | -0.01% | 0.01% | 0.00% | 0.02% |
|  | 17 | 17 (C) | 0.00% | 0.00% | 0.00% | 0.00% |
|  | 18 | 18 (C) | 0.03% | 0.02% | 0.03% | -0.01% |
|  | 19 | 19 (C) | 0.00% | 0.00% | 0.00% | 0.00% |
|  | 20 | 20 (C) | 0.00% | 0.00% | 0.00% | 0.01% |
|  | 21 | 21 (C) | 0.01% | 0.01% | 0.01% | 0.00% |
|  | 22 | 22 (C) | 0.01% | 0.02% | 0.01% | 0.00% |
|  | 23 | 23 (C) | 0.00% | 0.00% | 0.00% | 0.00% |
|  | 24 | 24 (C) | 0.00% | 0.01% | 0.01% | 0.00% |
|  | 25 | 25 (C) | 0.01% | 0.01% | 0.01% | 0.00% |
|  | 26 | 54 (C) | 6.85% | 6.74% | 6.80% | -0.11% |
|  | 27 | 55 (C) | 0.23% | 5.35% | 1.10% | 5.12% |
|  | 28 | 56 (C) | 5.93% | 1.11% | 2.57% | -4.81% |
|  | 29 | 57 (C) | 4.18% | 9.91% | 6.43% | 5.73% |
|  | 30 | 58 (C) | 2.40% | 2.92% | 2.65% | 0.52% |
|  | 31 | 59 (C) | 1.73% | 2.74% | 2.18% | 1.01% |
|  | 32 | 60 (C) | 12.56% | 0.69% | 2.95% | -11.87% |
|  | 33 | 61 (C) | 9.82% | 2.12% | 4.56% | -7.70% |

Table S4 (continued) Electron transfer contribution of atoms in TP-CF_3_-COOH+fentanyl S1, S2 and S3 process.

| Excited state | Heat map serial number | Atomic number | Hole | Electron | Overlap | Diff. |
| --- | --- | --- | --- | --- | --- | --- |
| S3 | 34 | 62 (C) | 9.87% | 2.11% | 4.56% | -7.76% |
|  | 35 | 63 (C) | 12.88% | 0.76% | 3.12% | -12.13% |
|  | 36 | 64 (C) | 0.84% | 1.73% | 1.21% | 0.88% |
|  | 37 | 65 (C) | 4.15% | 10.50% | 6.60% | 6.35% |
|  | 38 | 66 (C) | 0.81% | 2.16% | 1.32% | 1.34% |
|  | 39 | 67 (C) | 1.12% | 0.78% | 0.94% | -0.34% |
|  | 40 | 68 (C) | 0.19% | 0.27% | 0.23% | 0.08% |
|  | 41 | 69 (C) | 1.26% | 1.34% | 1.30% | 0.08% |
|  | 42 | 70 (C) | 0.14% | 0.71% | 0.32% | 0.57% |
|  | 43 | 71 (C) | 1.36% | 0.37% | 0.71% | -0.98% |
|  | 44 | 72 (C) | 2.84% | 2.84% | 2.84% | 0.00% |
|  | 45 | 73 (C) | 1.22% | 3.27% | 2.00% | 2.04% |
|  | 46 | 74 (C) | 6.67% | 7.32% | 6.99% | 0.66% |
|  | 47 | 75 (C) | 0.32% | 5.52% | 1.32% | 5.20% |
|  | 48 | 76 (C) | 5.06% | 1.50% | 2.75% | -3.56% |
|  | 49 | 77 (C) | 1.06% | 1.10% | 1.08% | 0.04% |
|  | 50 | 78 (C) | 0.24% | 0.34% | 0.29% | 0.10% |
|  | 51 | 79 (C) | 1.17% | 1.76% | 1.43% | 0.59% |
|  | 52 | 80 (C) | 0.16% | 1.05% | 0.41% | 0.89% |
|  | 53 | 81 (C) | 1.33% | 0.39% | 0.72% | -0.95% |
|  | 54 | 82 (C) | 0.18% | 5.55% | 1.00% | 5.37% |
|  | 55 | 83 (O) | 1.19% | 3.65% | 2.08% | 2.46% |
|  | 56 | 84 (O) | 0.22% | 1.16% | 0.50% | 0.94% |
|  | 57 | 85 (C) | 0.18% | 5.83% | 1.02% | 5.65% |
|  | 58 | 86 (O) | 1.13% | 3.87% | 2.09% | 2.75% |

Table S4 (continued). Electron transfer contribution of atoms in TP-CF_3_-COOH+fentanyl S1, S2 and S3 process.

| Excited state | Heat map serial number | Atomic number | Hole | Electron | Overlap | Diff. |
| --- | --- | --- | --- | --- | --- | --- |
| S3 | 59 | 87 (O) | 0.23% | 1.23% | 0.53% | 1.00% |
|  | 60 | 88 (C) | 0.07% | 0.20% | 0.11% | 0.13% |
|  | 61 | 89 (F) | 0.05% | 0.06% | 0.05% | 0.01% |
|  | 62 | 90 (F) | 0.02% | 0.02% | 0.02% | 0.00% |
|  | 63 | 91 (F) | 0.02% | 0.02% | 0.02% | 0.00% |
|  | 64 | 92 (C) | 0.06% | 0.29% | 0.13% | 0.23% |
|  | 65 | 93 (F) | 0.04% | 0.08% | 0.06% | 0.03% |
|  | 66 | 94 (F) | 0.02% | 0.04% | 0.03% | 0.02% |
|  | 67 | 95 (F) | 0.01% | 0.03% | 0.02% | 0.01% |
|  | Sum of hole shown above： | | 99.66% | Sum of electron shown above： | | 99.54% |

Table S5. The amount of electron transfer of TP-CF_3_-COOH+norfentanyl during electron excitation is calculated by IFCT method in Multiwfn.

| Excited  state | Fragment | Atom  indices | Each fragment  to hole | Each fragment  to electron | Transferred electrons  between fragments |
| --- | --- | --- | --- | --- | --- |
|  | 1 | 1-37 | 0.02% | 0.16% | 1 -> 2: 0.00000 |
| S1 (LE) |  |  |  |  | 1 <- 2: 0.00160 |
|  | 2 | 38-99 | 100.16% | 99.84% | Net 1 -> 2: -0.00160 |
|  | 1 | 1-37 | 75.07% | 0.76% | 1 -> 2: 0.74496 |
| S2 (IC) |  |  |  |  | 1 <- 2: 0.00189 |
|  | 2 | 38-99 | 24.93% | 99.24% | Net 1 -> 2: 0.74307 |
|  | 1 | 1-37 | 13.26% | 0.53% | 1 -> 2: 0.13193 |
| S3 (LE) |  |  |  |  | 1 <- 2: 0.00457 |
|  | 2 | 38-99 | 86.74% | 99.47% | Net 1 -> 2: 0.12735 |

Table S6. Electron transfer contribution of atoms in TP-CF_3_-COOH+norfentanyl S1, S2 and S3 process.

| Excited state | Heat map serial number | Atomic number | Hole | Electron | Overlap | Diff. |
| --- | --- | --- | --- | --- | --- | --- |
| S1 | 1 | 1 (C) | 0.00% | 0.00% | 0.00% | 0.00% |
|  | 2 | 2 (C) | 0.00% | 0.00% | 0.00% | 0.00% |
|  | 3 | 3 (C) | 0.00% | 0.00% | 0.00% | 0.00% |
|  | 4 | 4 (O) | 0.00% | 0.00% | 0.00% | 0.00% |
|  | 5 | 5 (N) | 0.00% | 0.00% | 0.00% | 0.00% |
|  | 6 | 6 (C) | 0.00% | 0.00% | 0.00% | 0.00% |
|  | 7 | 7 (C) | 0.00% | 0.00% | 0.00% | 0.00% |
|  | 8 | 8 (C) | 0.00% | 0.01% | 0.00% | 0.01% |
|  | 9 | 9 (N) | 0.00% | 0.01% | 0.00% | 0.01% |
|  | 10 | 10 (C) | 0.00% | 0.00% | 0.00% | 0.00% |
|  | 11 | 11 (C) | 0.00% | 0.00% | 0.00% | 0.00% |
|  | 12 | 12 (C) | 0.00% | 0.00% | 0.00% | 0.00% |
|  | 13 | 13 (C) | -0.04% | 0.01% | 0.00% | 0.04% |
|  | 14 | 14 (C) | -0.04% | 0.01% | 0.00% | 0.05% |
|  | 15 | 15 (C) | 0.00% | 0.00% | 0.00% | 0.00% |
|  | 16 | 16 (C) | -0.04% | 0.00% | 0.00% | 0.04% |
|  | 17 | 17 (C) | -0.05% | 0.00% | 0.00% | 0.05% |
|  | 18 | 37 (C) | 4.40% | 4.64% | 4.52% | 0.24% |
|  | 19 | 38 (C) | 0.17% | 1.74% | 0.55% | 1.57% |
|  | 20 | 39 (C) | 3.79% | 2.69% | 3.19% | -1.10% |
|  | 21 | 40 (C) | 2.38% | 4.64% | 3.32% | 2.27% |
|  | 22 | 41 (C) | 3.47% | 3.52% | 3.49% | 0.05% |
|  | 23 | 42 (C) | 0.28% | 1.00% | 0.53% | 0.72% |
|  | 24 | 43 (C) | 16.10% | 12.70% | 14.30% | -3.40% |

Table S6 (continued). Electron transfer contribution of atoms in TP-CF_3_-COOH+norfentanyl S1, S2 and S3 process.

| Excited state | Heat map serial number | Atomic number | Hole | Electron | Overlap | Diff. |
| --- | --- | --- | --- | --- | --- | --- |
| S1 | 25 | 44 (C) | 11.13% | 10.21% | 10.66% | -0.92% |
|  | 26 | 45 (C) | 11.19% | 9.38% | 10.24% | -1.82% |
|  | 27 | 46 (C) | 15.78% | 13.10% | 14.38% | -2.68% |
|  | 28 | 47 (C) | 1.28% | 0.84% | 1.04% | -0.44% |
|  | 29 | 48 (C) | 2.47% | 4.07% | 3.17% | 1.60% |
|  | 30 | 49 (C) | 1.33% | 0.75% | 1.00% | -0.58% |
|  | 31 | 50 (C) | 1.93% | 1.14% | 1.48% | -0.79% |
|  | 32 | 51 (C) | 0.10% | 0.52% | 0.23% | 0.42% |
|  | 33 | 52 (C) | 1.85% | 1.61% | 1.73% | -0.24% |
|  | 34 | 53 (C) | 0.10% | 0.11% | 0.11% | 0.01% |
|  | 35 | 54 (C) | 1.89% | 1.66% | 1.77% | -0.23% |
|  | 36 | 55 (C) | 3.41% | 3.33% | 3.37% | -0.08% |
|  | 37 | 56 (C) | 0.28% | 0.88% | 0.50% | 0.60% |
|  | 38 | 57 (C) | 4.33% | 4.30% | 4.32% | -0.03% |
|  | 39 | 58 (C) | 0.15% | 1.51% | 0.48% | 1.36% |
|  | 40 | 59 (C) | 3.72% | 2.68% | 3.16% | -1.05% |
|  | 41 | 60 (C) | 1.77% | 1.04% | 1.35% | -0.73% |
|  | 42 | 61 (C) | 0.10% | 0.53% | 0.23% | 0.43% |
|  | 43 | 62 (C) | 1.83% | 1.47% | 1.64% | -0.36% |
|  | 44 | 63 (C) | 0.09% | 0.10% | 0.10% | 0.01% |
|  | 45 | 64 (C) | 1.90% | 1.64% | 1.76% | -0.26% |
|  | 46 | 65 (C) | 0.20% | 1.80% | 0.59% | 1.60% |
|  | 47 | 66 (O) | 0.68% | 1.41% | 0.98% | 0.73% |
|  | 48 | 67 (O) | 0.16% | 0.47% | 0.28% | 0.31% |

Table S6 (continued). Electron transfer contribution of atoms in TP-CF_3_-COOH+norfentanyl S1, S2 and S3 process.

| Excited state | Heat map serial number | Atomic number | Hole | Electron | Overlap | Diff. |
| --- | --- | --- | --- | --- | --- | --- |
|  | 49 | 68 (C) | 0.17% | 1.55% | 0.51% | 1.38% |
|  | 50 | 69 (O) | 0.69% | 1.32% | 0.95% | 0.63% |
|  | 51 | 70 (O) | 0.15% | 0.41% | 0.24% | 0.26% |
|  | 52 | 71 (C) | 0.10% | 0.16% | 0.13% | 0.06% |
|  | 53 | 72 (F) | 0.07% | 0.05% | 0.06% | -0.02% |
|  | 54 | 73 (F) | 0.02% | 0.02% | 0.02% | 0.00% |
|  | 55 | 74 (F) | 0.02% | 0.03% | 0.03% | 0.00% |
| S1 | 56 | 75 (C) | 0.09% | 0.17% | 0.13% | 0.07% |
|  | 57 | 76 (F) | 0.06% | 0.05% | 0.06% | -0.01% |
|  | 58 | 77 (F) | 0.02% | 0.01% | 0.01% | 0.00% |
|  | 59 | 78 (F) | 0.04% | 0.03% | 0.04% | -0.01% |
|  | Sum of hole shown above | | 99.54% | Sum of electron shown above | | 99.33% |
|  | 1 | 1 (C) | 0.01% | 0.00% | 0.00% | 0.00% |
|  | 2 | 2 (C) | 0.01% | 0.00% | 0.00% | -0.01% |
|  | 3 | 3 (C) | 0.06% | 0.00% | 0.01% | -0.05% |
| S2 | 4 | 4 (O) | 0.05% | 0.00% | 0.00% | -0.05% |
|  | 5 | 5 (N) | 0.31% | 0.00% | 0.02% | -0.31% |
|  | 6 | 6 (C) | 0.04% | 0.00% | 0.00% | -0.04% |
|  | 7 | 7 (C) | 1.29% | 0.01% | 0.10% | -1.28% |
|  | 8 | 8 (C) | 3.10% | 0.03% | 0.29% | -3.07% |
|  | 9 | 9 (N) | 51.09% | 0.04% | 1.49% | -51.04% |
|  | 10 | 10 (C) | 2.73% | 0.01% | 0.16% | -2.72% |
|  | 11 | 11 (C) | 0.78% | 0.01% | 0.06% | -0.78% |

Table S6 (continued). Electron transfer contribution of atoms in TP-CF_3_-COOH+norfentanyl S1, S2 and S3 process.

| Excited state | Heat map serial number | Atomic number | Hole | Electron | Overlap | Diff. |
| --- | --- | --- | --- | --- | --- | --- |
|  | 12 | 12 (C) | 0.80% | 0.00% | 0.00% | -0.80% |
|  | 13 | 13 (C) | 0.96% | 0.02% | 0.14% | -0.94% |
|  | 14 | 14 (C) | 0.12% | 0.03% | 0.06% | -0.09% |
|  | 15 | 15 (C) | 0.34% | 0.00% | 0.03% | -0.34% |
|  | 16 | 16 (C) | 0.52% | 0.01% | 0.07% | -0.51% |
|  | 17 | 17 (C) | 0.05% | 0.01% | 0.02% | -0.04% |
|  | 18 | 37 (C) | 1.77% | 10.95% | 4.40% | 9.18% |
| S2 | 19 | 38 (C) | 0.87% | 7.49% | 2.56% | 6.61% |
|  | 20 | 39 (C) | 4.14% | 2.85% | 3.44% | -1.29% |
|  | 21 | 40 (C) | 0.90% | 11.99% | 3.28% | 11.09% |
|  | 22 | 41 (C) | 0.98% | 3.70% | 1.91% | 2.71% |
|  | 23 | 42 (C) | 3.36% | 5.65% | 4.35% | 2.29% |
|  | 24 | 43 (C) | 2.04% | 4.93% | 3.17% | 2.89% |
|  | 25 | 44 (C) | 1.76% | 7.51% | 3.64% | 5.75% |
|  | 26 | 45 (C) | 1.29% | 2.64% | 1.84% | 1.35% |
|  | 27 | 46 (C) | 1.97% | 5.75% | 3.36% | 3.78% |
|  | 28 | 47 (C) | 0.17% | 0.70% | 0.35% | 0.53% |
|  | 29 | 48 (C) | 0.40% | 1.74% | 0.84% | 1.34% |
|  | 30 | 49 (C) | 0.03% | 0.29% | 0.09% | 0.26% |
|  | 31 | 50 (C) | 0.21% | 0.54% | 0.34% | 0.33% |
|  | 32 | 51 (C) | 0.06% | 0.23% | 0.11% | 0.18% |
|  | 33 | 52 (C) | 0.27% | 0.80% | 0.46% | 0.54% |
|  | 34 | 53 (C) | 0.09% | 0.29% | 0.16% | 0.20% |
|  | 35 | 54 (C) | 0.32% | 0.62% | 0.45% | 0.30% |

Table S6 (continued). Electron transfer contribution of atoms in TP-CF_3_-COOH+norfentanyl S1, S2 and S3 process.

| Excited state | Heat map serial number | Atomic number | Hole | Electron | Overlap | Diff. |
| --- | --- | --- | --- | --- | --- | --- |
|  | 36 | 55 (C) | 0.40% | 1.17% | 0.68% | 0.77% |
|  | 37 | 56 (C) | 0.11% | 0.58% | 0.26% | 0.47% |
|  | 38 | 57 (C) | 0.72% | 1.85% | 1.15% | 1.12% |
|  | 39 | 58 (C) | 0.01% | 0.78% | 0.06% | 0.77% |
|  | 40 | 59 (C) | 0.56% | 1.00% | 0.75% | 0.44% |
|  | 41 | 60 (C) | 0.16% | 0.41% | 0.25% | 0.25% |
|  | 42 | 61 (C) | 0.01% | 0.28% | 0.06% | 0.27% |
| S2 | 43 | 62 (C) | 0.10% | 0.70% | 0.27% | 0.60% |
|  | 44 | 63 (C) | 0.02% | 0.08% | 0.04% | 0.06% |
|  | 45 | 64 (C) | 0.18% | 0.66% | 0.34% | 0.49% |
|  | 46 | 65 (C) | 0.08% | 11.50% | 0.93% | 11.43% |
|  | 47 | 66 (O) | 1.54% | 6.88% | 3.26% | 5.34% |
|  | 48 | 67 (O) | 0.13% | 2.44% | 0.57% | 2.30% |
|  | 49 | 68 (C) | 0.02% | 0.78% | 0.13% | 0.76% |
|  | 50 | 69 (O) | 0.13% | 0.63% | 0.29% | 0.50% |
|  | 51 | 70 (O) | 0.02% | 0.20% | 0.07% | 0.17% |
|  | 52 | 71 (C) | 0.01% | 0.13% | 0.04% | 0.12% |
|  | 53 | 72 (F) | 0.01% | 0.03% | 0.02% | 0.02% |
|  | 54 | 73 (F) | 0.00% | 0.02% | 0.01% | 0.01% |
|  | 55 | 74 (F) | 0.00% | 0.02% | 0.01% | 0.01% |
|  | 56 | 75 (C) | 0.01% | 0.10% | 0.03% | 0.09% |
|  | 57 | 76 (F) | 0.00% | 0.03% | 0.01% | 0.02% |
|  | 58 | 77 (F) | 0.00% | 0.01% | 0.00% | 0.01% |
|  | 59 | 78 (F) | 0.00% | 0.02% | 0.01% | 0.01% |

Table S6 (continued). Electron transfer contribution of atoms in TP-CF_3_-COOH+norfentanyl S1, S2 and S3 process.

| Excited state | Heat map serial number | Atomic number | Hole | Electron | Overlap | Diff. |
| --- | --- | --- | --- | --- | --- | --- |
| S2 | Sum of hole shown above | | 87.09% | Sum of electron shown above | | 99.10% |
|  | 1 | 1 (C) | 0.00% | 0.00% | 0.00% | 0.00% |
|  | 2 | 2 (C) | 0.01% | 0.00% | 0.00% | -0.01% |
|  | 3 | 3 (C) | 0.01% | 0.00% | 0.00% | -0.02% |
|  | 4 | 4 (O) | 0.04% | 0.00% | 0.00% | -0.04% |
|  | 5 | 5 (N) | 0.04% | 0.00% | 0.00% | -0.04% |
|  | 6 | 6 (C) | 0.01% | 0.00% | 0.00% | -0.01% |
| S3 | 7 | 7 (C) | 0.20% | 0.01% | 0.04% | -0.19% |
|  | 8 | 8 (C) | 0.54% | 0.04% | 0.14% | -0.50% |
|  | 9 | 9 (N) | 9.05% | 0.03% | 0.50% | -9.03% |
|  | 10 | 10 (C) | 0.45% | 0.01% | 0.05% | -0.45% |
|  | 11 | 11 (C) | 0.13% | 0.00% | 0.02% | -0.12% |
|  | 12 | 12 (C) | 0.18% | 0.00% | 0.03% | -0.18% |
|  | 13 | 13 (C) | 0.19% | 0.02% | 0.06% | -0.18% |
|  | 14 | 14 (C) | 0.01% | 0.01% | 0.01% | 0.00% |
|  | 15 | 15 (C) | 0.07% | 0.00% | 0.01% | -0.06% |
|  | 16 | 16 (C) | 0.09% | 0.00% | 0.02% | -0.09% |
|  | 17 | 17 (C) | -0.04% | 0.00% | 0.02% | 0.04% |
|  | 18 | 37 (C) | 5.53% | 8.74% | 6.95% | 3.21% |
|  | 19 | 38 (C) | 1.53% | 4.05% | 2.49% | 2.52% |
|  | 20 | 39 (C) | 2.42% | 3.16% | 2.76% | 0.75% |
|  | 21 | 40 (C) | 3.42% | 12.63% | 6.57% | 9.21% |
|  | 22 | 41 (C) | 5.49% | 1.89% | 3.23% | -3.60% |

Table S6 (continued). Electron transfer contribution of atoms in TP-CF_3_-COOH+norfentanyl S1, S2 and S3 process.

| Excited state | Heat map serial number | Atomic number | Hole | Electron | Overlap | Diff. |
| --- | --- | --- | --- | --- | --- | --- |
|  | 23 | 42 (C) | 0.25% | 6.76% | 1.29% | 6.51% |
|  | 24 | 43 (C) | 12.25% | 1.70% | 4.56% | -10.55% |
|  | 25 | 44 (C) | 10.02% | 2.66% | 5.16% | -7.35% |
|  | 26 | 45 (C) | 7.70% | 2.20% | 4.12% | -5.49% |
|  | 27 | 46 (C) | 11.39% | 1.51% | 4.14% | -9.88% |
|  | 28 | 47 (C) | 1.17% | 3.14% | 1.92% | 1.97% |
| S3 | 29 | 48 (C) | 2.57% | 6.86% | 4.20% | 4.29% |
|  | 30 | 49 (C) | 0.76% | 0.81% | 0.79% | 0.05% |
|  | 31 | 50 (C) | 1.50% | 0.99% | 1.22% | -0.51% |
|  | 32 | 51 (C) | 0.19% | 1.02% | 0.44% | 0.83% |
|  | 33 | 52 (C) | 1.69% | 2.61% | 2.10% | 0.92% |
|  | 34 | 53 (C) | 0.18% | 0.68% | 0.35% | 0.50% |
|  | 35 | 54 (C) | 1.61% | 1.26% | 1.43% | -0.35% |
|  | 36 | 55 (C) | 2.39% | 1.73% | 2.04% | -0.66% |
|  | 37 | 56 (C) | 0.56% | 2.45% | 1.17% | 1.90% |
|  | 38 | 57 (C) | 4.40% | 4.84% | 4.61% | 0.44% |
|  | 39 | 58 (C) | 0.23% | 2.94% | 0.82% | 2.71% |
|  | 40 | 59 (C) | 3.07% | 1.41% | 2.08% | -1.66% |
|  | 41 | 60 (C) | 1.03% | 0.30% | 0.56% | -0.73% |
|  | 42 | 61 (C) | 0.06% | 0.21% | 0.11% | 0.15% |
|  | 43 | 62 (C) | 1.16% | 0.61% | 0.84% | -0.55% |
|  | 44 | 63 (C) | 0.07% | 0.16% | 0.11% | 0.08% |
|  | 45 | 64 (C) | 1.18% | 0.39% | 0.68% | -0.78% |
|  | 46 | 65 (C) | 0.21% | 7.75% | 1.27% | 7.54% |

Table S6 (continued). Electron transfer contribution of atoms in TP-CF_3_-COOH+norfentanyl S1, S2 and S3 process.

| Excited state | Heat map serial number | Atomic number | Hole | Electron | Overlap | Diff. |
| --- | --- | --- | --- | --- | --- | --- |
|  | 47 | 66 (O) | 0.72% | 4.69% | 1.84% | 3.97% |
|  | 48 | 67 (O) | 0.31% | 1.71% | 0.73% | 1.40% |
|  | 49 | 68 (C) | 0.13% | 3.53% | 0.68% | 3.39% |
|  | 50 | 69 (O) | 0.68% | 2.38% | 1.27% | 1.70% |
|  | 51 | 70 (O) | 0.18% | 0.77% | 0.37% | 0.59% |
|  | 52 | 71 (C) | 0.09% | 0.39% | 0.18% | 0.31% |
| S3 | 53 | 72 (F) | 0.07% | 0.12% | 0.09% | 0.05% |
|  | 54 | 73 (F) | 0.03% | 0.04% | 0.03% | 0.02% |
|  | 55 | 74 (F) | 0.03% | 0.05% | 0.04% | 0.02% |
|  | 56 | 75 (C) | 0.06% | 0.07% | 0.07% | 0.01% |
|  | 57 | 76 (F) | 0.04% | 0.02% | 0.03% | -0.02% |
|  | 58 | 77 (F) | 0.01% | 0.01% | 0.01% | -0.01% |
|  | 59 | 78 (F) | 0.03% | 0.02% | 0.02% | -0.01% |
|  | Sum of hole shown above | | 97.34% | Sum of electron shown above | | 99.38% |

Table S7. The amount of electron transfer of TP-CF_3_-COOH+3-methylfentanyl during electron excitation is calculated by IFCT method in Multiwfn.

| Excited  state | Fragment | Atom   indices | Each fragment  to hole | Each fragment   to electron | Transferred electrons  between fragments |
| --- | --- | --- | --- | --- | --- |
| S1 (LE) | 1 | 1-51, 114-118 | 0.02% | 0.23% | 1 -> 2: 0.00024 |
|  |  |  |  |  | 1 <- 2: 0.00225 |
|  | 2 | 52-113 | 99.98% | 99.77% | Net 1 -> 2: -0.00201 |
| S2 (IC) | 1 | 1-51, 114-118 | 99.00% | 0.56% | 1 -> 2: 0.98446 |
|  |  |  |  |  | 1 <- 2: 0.00006 |
|  | 2 | 52-113 | 1.00% | 99.44% | Net 1 -> 2: 0.98440 |
| S3 (LE) | 1 | 1-51, 114-118 | 0.06% | 0.37% | 1 -> 2: 0.00057 |
|  |  |  |  |  | 1 <- 2: 0.00366 |
|  | 2 | 52-113 | 99.94% | 99.94% | Net 1 -> 2: -0.00309 |

Table S8. The amount of electron transfer of TP-CF_3_-COOH+ocfentanyl during electron excitation is calculated by IFCT method in Multiwfn.

| Excited  state | Fragment | Atom   indices | Each fragment  to hole | Each fragment   to electron | Transferred electrons  between fragments |
| --- | --- | --- | --- | --- | --- |
| S1 (LE) | 1 | 1-48, 111-116 | 0.04% | 0.20% | 1 -> 2: 0.00043 |
|  |  |  |  |  | 1 <- 2: 0.00199 |
|  | 2 | 49-110 | 99.96% | 99.80% | Net 1->2: -0.00156 |
| S2 (IC) | 1 | 1-48, 111-116 | 98.82% | 0.56% | 1 -> 2: 0.98263 |
|  |  |  |  |  | 1 <- 2: 0.00007 |
|  | 2 | 49-110 | 1.18% | 99.44% | Net 1->2: 0.98256 |
| S3 (LE) | 1 | 1-48, 111-116 | 0.00% | 0.44% | 1 -> 2: 0.00002 |
|  |  |  |  |  | 1 <- 2: 0.00439 |
|  | 2 | 49-110 | 100.00% | 99.56% | Net 1 -> 2: -0.00438 |

Table S9. Determination of norfentanyl (μg/L) using the developed method and HPLC-MS/MS.

| Spiked  (μg /L ) | This method | |  | HPLC-MS/MS | |
| --- | --- | --- | --- | --- | --- |
|  | Determined  (mean ± SD, n = 6) | RSD (%) |  | Determined  (mean ± SD, n = 6) | RSD (%) |
| 0 | — | — |  | — | — |
| 1 | 0.98±0.02 | 1.6 |  | 1.02±0.01 | 0.8 |
| 100 | 99.59±2.1 | 2.1 |  | 102.46±1.2 | 1.2 |
| 1000 | 1036.68±13 | 1.3 |  | 1041.14±10 | 1.0 |
| “—” means not found. | | | | | |

Table S10. Determination of fentanyl (μg/L) using the developed method and HPLC-MS/MS.

| Spiked (μg /L) | This method | |  | HPLC-MS/MS | |
| --- | --- | --- | --- | --- | --- |
|  | Determined  (mean ± SD, n = 6) | RSD (%) |  | Determined  (mean ± SD, n = 6) | RSD (%) |
| 0 | — | — |  | — | — |
| 1 | 0.99±0.02 | 1.5 |  | 0.99±0.01 | 1.2 |
| 100 | 100.13±1.7 | 1.7 |  | 101.19±1.1 | 1.1 |
| 1000 | 999.33±19.4 | 1.9 |  | 1000.12±22.9 | 2.3 |
| “—” means not found. | | | | | |

Table S11. Comparison of different methods for the determination of fentanyl.

| Analytical methods | LOD(μg/L) | Detection time(s) | Ref. |
| --- | --- | --- | --- |
| UHPLC-MS/MS | 30 | 480 | 47 |
| MOI-MS/MS | 3 | 144 | 48 |
| E-3D-SenS | 5.72 | 200 | 49 |
| SQDS | 2.66 | 120 | 50 |
| g-C_3_N_4_-PANI/GCE | 2.02 | 78 | 51 |
| TP-CF_3_-COOH | 0.0002 | 60 | This work |

UHPLC-MS/MS: ultrahigh-performance liquid chromatography coupled to tandem mass spectrometry, MOI-MS/MS: Microfluidic Open Interfacecoupled to tandem mass spectrometry, E-3D-SenS: Electrochemical 3D-printed Sensing Set, SQDS: Supramolecular–Quantum Dot System, g-C_3_N_4_-PANI/GCE: g-C_3_N_4_ and polyaniline biosensor.

**References**

47.	Nan, Q.; Ping, X.; Baohua, S.; Xianyi, Z.; Yan, S.; Song, FY. Journal of Chromatography B 2019, 1124 (January), 82-99, DOI: 10.1016/j.jchromb.2019.05.025

48.	Nazdrajic, E; Rickert, DA.; Pawliszyn, J. Analytical Chemistry 2024, 96 (2), 821-827 DOI: 10.1021/acs.analchem.3c04354

49.	Conrado, TT.; Pedao, ER.; Valdir, SF.; Katia, M.; Lucca, BG. Sensors and Actuators: B. Chemical 2025, 427, 137215 DOI: 10.1016/j.snb.2024.137215

50.	Gao, YJ; Shirinichi, F; Audrey, H.; Runyao, Z.; Wang, YC. Small 2024, 2407702. DOI: 10.1002/smll.202407702

51.	Hu, GY.; Li, HX.; Liu, F. Alexandria Engineering Journal 2024, 87, 515-523 DOI: 10.1016/j.aej.2023.12.043
